# Combinatorial Clustering of Residue Position Subsets Predicts Inhibitor Affinity across the Human Kinome

**DOI:** 10.1371/journal.pcbi.1003087

**Published:** 2013-06-06

**Authors:** Drew H. Bryant, Mark Moll, Paul W. Finn, Lydia E. Kavraki

**Affiliations:** 1 Department of Computer Science, Rice University, Houston, Texas, United States of America; 2 InhibOx Ltd, Oxford, United Kingdom; 3 Department of Bioengineering, Rice University, Houston, Texas, United States of America; Princeton University, United States of America

## Abstract

The protein kinases are a large family of enzymes that play fundamental roles in propagating signals within the cell. Because of the high degree of binding site similarity shared among protein kinases, designing drug compounds with high specificity among the kinases has proven difficult. However, computational approaches to comparing the 3-dimensional geometry and physicochemical properties of key binding site residue positions have been shown to be informative of inhibitor selectivity. The Combinatorial Clustering Of Residue Position Subsets (ccorps) method, introduced here, provides a semi-supervised learning approach for identifying structural features that are correlated with a given set of annotation labels. Here, ccorps is applied to the problem of identifying structural features of the kinase atp binding site that are informative of inhibitor binding. ccorps is demonstrated to make perfect or near-perfect predictions for the binding affinity profile of 8 of the 38 kinase inhibitors studied, while only having overall poor predictive ability for 1 of the 38 compounds. Additionally, ccorps is shown to identify shared structural features across phylogenetically diverse groups of kinases that are correlated with binding affinity for particular inhibitors; such instances of structural similarity among phylogenetically diverse kinases are also shown to not be rare among kinases. Finally, these function-specific structural features may serve as potential starting points for the development of highly specific kinase inhibitors.

## Author Summary

The kinases are a group of essential signaling proteins within the cell and are the largest family of enzymes encoded by the human genome. The high degree of binding site similarity shared across the protein kinases has made them difficult targets for which to design highly selective inhibitors, but kinome-wide binding site analysis can help predict unintended off-target inhibitions. Given the increasingly large number of available kinase structures, kinome-wide comparative analysis of binding sites is now possible. In this paper, the Combinatorial Clustering Of Residue Position Subsets (ccorps) method is introduced and used to synthesize kinome-wide structure datasets with a kinome-wide inhibitor affinity screening dataset consisting of 38 kinase inhibitors. ccorps identifies structural features of the kinase binding site that are correlated with an inhibitor binding and uses these features to predict if this inhibitor will be capable of binding to uncharacterized kinases. This paper demonstrates the ability of ccorps to accurately predict inhibitor binding and identify features of the kinase binding site that are unique to kinases capable of binding a given inhibitor.

## Introduction

The protein kinases constitute the largest enzyme family encoded by the human genome, with currently 518 known sequences, making up 1.7% of all human genes [1], [2]. Because these protein kinases are intimately involved in cellular communication and regulation networks, the loss of normal kinase regulation has been implicated in a wide variety of pathological conditions. The large number of disease states found to be associated with kinase dysregulation has motivated the development of kinase-specific inhibitor compounds and research to discover protein kinase inhibitors has come to constitute 20–30% of the drug development programs at many companies [1].

The bulk of this effort has been directed at identifying inhibitors that bind at the atp binding site. However, due to the large number of existing protein kinase domains and the high degree of (atp) binding site similarity among them, designing highly selective inhibitors has proven difficult. For example, type I kinase inhibitors that only target the atp site have typically been found to have low selectivity across the kinome [3]. To increase inhibitor selectivity, type II inhibitors bind both the atp site and the immediately adjacent allosteric site. By also binding to the allosteric site, type II inhibitors are able to make additional highly specific interactions, thereby allowing them to be more selective [3].

Identifying highly specific structural features that can be uniquely targeted by inhibitors can be facilitated by comparative analysis of multiple kinase structures [4]. Comparative analysis of multiple structures allows for the identification of kinase structural features that are available for inhibitor targeting as well as insight into the effect of activation conformation dynamics, such as structural features that are only available for targeting in the inactive, DFG-out conformation [3]–[6]. Furthermore, combining structure and sequence is important when analyzing the kinases holistically due to the large degree of sequence divergence among the protein kinases [7]. A specific example of the insight derived from the comparative analysis of kinase structural features follows.

Many of the effective inhibitor selectivity strategies involve exploiting the differences in the size of the atp binding site and targeting residue variability at a few key positions [3], [8]. These structure-based comparison approaches have proven more useful than sequence-only measures of overall kinase similarity in evaluating the potential selectivity profile of inhibitors [8]. For example, the size of the gatekeeper residue directly moderates the availability of a hydrophobic pocket. Inhibitors having larger functional groups that bind this hydrophobic pocket may be able to select for the roughly 20% of protein kinases that have a relatively small gatekeeper residue (e.g., Gly, Val, Ala or Thr). This is because kinases with a larger gatekeeper residue (e.g., Phe) do not have a large enough hydrophobic pocket to accommodate the inhibitor [8]. However, in order to select for an even more specific subset of the human kinome, it has proven necessary to take advantage of multiple structural features of the kinase binding site (both atp and allosteric sites) simultaneously [3], [8].

A review of related work is given below. Recent work has illustrated that local structural similarity exists among phylogenetically diverse groups of kinases [5], [9] and has highlighted the importance of large-scale, multiple-structure analysis of structure-affinity relationships among the kinases [9], [10].

The PharmMap method [10] incorporates an aligned set of receptor-ligand co-crystals in order to identify pharmacophores common to a set of inhibitors. It has been developed to identify kinase inhibitor pharmacophores useful for selecting molecules for kinase screening panels.

Huang et al. have utilized a knowledge-based approach to constructing a minimal binding site “fingerprint” that captures only a pre-specified set of well-studied, structurally selective features, such as the size and hydrogen-bonding ability of the gatekeeper residue [8]. The per-kinase fingerprint utilizes nine binding site features (e.g., residue type at gatekeeper position) that have been shown to encode for selectivity among type I inhibitors. Anecdotally, kinases with similar fingerprints were shown to also have similar inhibitor selectivity profiles [8], illustrating the utility of structural features in predicting and understanding kinase selectivity.

Rather than relying upon pre-specified structural features, the recently developed Pocketfeature method decomposes a binding site into all possible “micro-environments” [11]. Pairs of kinase binding sites with highly similar sets of micro-environments were anecdotally shown to share a common inhibitor in 9 out of the top 50 most similar (as calculated by Pocketfeature) kinase binding site pairs. The CavBase [12] cavity matching approach has been used to cluster kinase atp binding cavities from multiple families across the kinome [5]. The kinase binding cavity clusterings derived from CavBase have been shown to generally agree with the sequence-derived kinase families and sub-families [5], demonstrating that the overall kinase cavity is well-conserved within families.

Recent work by Jackson et al. demonstrated a related structural clustering approach to predicting kinase inhibitor binding affinities [9]. Their geometric hashing approach to whole-site comparison of the atp binding pocket was demonstrated to be effective at identifying possible instances of inhibitor cross-reactivity and further emphasized the importance of taking into account subtle conformational changes in the binding site.

However, despite the successes of existing approaches, several outstanding problems to identifying structural features of the kinase binding site that are predictive of inhibitor selectivity remain. The reliance upon a single, representative structure precludes the ability of existing methods to identify features common only to active conformations if an inactive structure is chosen as representative (and vice versa). Additionally, choosing one representative structure disregards the role that binding site flexibility and plasticity may play in inhibitor interactions. Furthermore, the availability of multiple structures for *individual* kinases, exhibiting a variety of binding site conformations and bound ligands, provides a vast quantity of structure data that remains unexploited by existing approaches. Much of the difficulty in incorporating multiple conformations per individual kinase sequence into existing analyses stems from the non-uniform distribution of available kinase structures, with kinases such as CDK2 having more than a hundred available crystallographic structures while other kinases have only a single (or no) available structure. Finally, the availability of multiple kinase structures known *to bind* a given inhibitor and other kinase binding sites known *not to bind* that same inhibitor provides a rich set of structural examples and counter-examples beyond a single instance of pairwise similarity. Existing receptor-based methods focus on identifying meaningful pairwise similarity to a characterized kinase known to bind a given inhibitor. These methods currently do not account for the similarity of a given kinase binding site to other kinase sites that have been characterized *to not bind* the inhibitor in question.

To this end we have developed the Combinatorial Clustering Of Residue Position Subsets (ccorps) method. ccorps solves the following problem: given a set of sequence-aligned kinase domains (each having 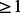 available PDB structures) and a per-sequence inhibitor binding *label* (either **binds, does-not-bind or unknown**), predict whether a given kinase domain binds the given inhibitor. Taking a set of kinase binding site residue positions as input, ccorps identifies clusters of kinases that share structurally and chemically similar subsets of residue positions. Given a particular kinase with unknown ability to bind a particular inhibitor, ccorps identifies kinase binding sites that share similar residue positions that are both known *to bind* and *not to bind* the inhibitor (i.e., it finds evidence both *for* and *against* binding a particular inhibitor). Finally, ccorps aggregates the residue position subset similarities for all possible 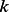-position subsets of the kinase binding site and predicts whether or not the given inhibitor will bind the given uncharacterized kinase binding site.

In addition to the development of ccorps, three major results from the analysis of the human kinome are presented here. First, the identification of structural features within the kinase atp binding site that are correlated with the ability of certain kinases to bind specific inhibitors is demonstrated. Second, the existence of affinity-correlated structural features that are shared among kinases from distinct families of the kinome are enumerated, shown to be not rare and also to differ depending upon the inhibitor being analyzed. Third, the ability of ccorps to predict the affinity for kinases lacking affinity annotations is quantified and compared to a recent structural binding site analysis approach [9].

ccorps is demonstrated to make perfect or near-perfect predictions for the binding ability of 8 of the 38 kinase inhibitors studied, while only having overall poor predictive ability for 1 of the 38 compounds. The performance of ccorps for predicting inhibitor binding is compared to the method of Jackson et al. [9] and shown to meet or exceed the predictive ability for the subset of the 38 inhibitors also tested by Jackson et al. We also compare ccorps against a sequence-based approach and show that they have complementary strengths. Finally, ccorps is shown to identify shared structural features across phylogenetically diverse groups of kinases that are correlated with binding affinity for particular inhibitors; such instances of structural similarity among phylogenetically diverse kinases are also shown to not be rare among kinases. These function-specific structural features may serve as potential starting points for the development of highly specific kinase inhibitors and provide a basis for understanding patterns of inhibition by compounds such as sunitinib that target multiple kinases [13].

In contrast to existing pairwise binding site comparison approaches, ccorps provides an automated way to incorporate the similarity of an uncharacterized binding site to *all* characterized binding site structures, *without* the need to manually select a reference binding site. ccorps also accounts for the similarity of an uncharacterized binding site to both kinases that *bind* and those that *do not bind* a particular inhibitor.

The high degree of atp binding site similarity shared across the protein kinases has made them a difficult target for which to design highly selective inhibitors. However, by identifying the patterns of local structural similarity among binding sites at the kinome scale, potential off-target interactions may be identifiable at earlier stages of pharmaceutical development and compensated for by further inhibitor modification. This would allow researchers to make predictions of binding affinity for a given ligand across the kinome with less experimental data. Furthermore, the emergence of kinase inhibitor resistance due to binding site position mutations may be better understood through the identification of kinases having similar structural features at the mutated positions. Structural features that are found to be unique to one or a small number of chosen kinases may provide the starting point for designing highly specific inhibitor interactions and therefore highly selective protein kinase inhibitors.

## Methods

The Combinatorial Clustering Of Residue Position Subsets (ccorps) method is designed to solve a very general semi-supervised learning problem:

*Find the structural features among the set of proteins that are correlated with a particular set of annotation labels.*

While in the *Results* section we focus on the specific problem of predicting ligand binding affinity across the human kinome, we will first describe ccorps in its most general form. To solve the general semi-supervised learning problem stated above, ccorps requires the following interface:

**Input:** an aligned set of protein substructures, where a substructure is defined as a collection of residues not necessarily contiguous in sequence but grouped together in 3D.

**Input:** annotation labels for *some* of the protein substructures.

**Output:** predicted annotation labels for the *unlabeled* protein substructures.

The per-substructure annotation labels may derived from a wide range of sources [14]–[25]. While this paper focuses on the application of ccorps for the prediction of inhibitor binding affinity annotations for the human kinome, ccorps generalizes to a variety of annotation prediction problems. The ability of ccorps to also identify specificity-determining enzymatic substructures for the prediction of ec class annotations for 48 different protein families is outlined in *Text S2* and summarized at the end of this section.

### Combinatorial clustering of residue position subsets

In order to identify locally similar features among substructures, all 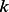-sized combinations of the 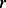 residue positions (i.e., 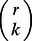 combinations) are generated. For example, given 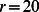 and 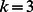, all 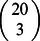 3-position subsets (1140 subsets) are generated. Then, each of these position subsets are examined one-by-one. Continuing the example, given the position subset 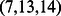, all the protein structures are compared by examining the pairwise similarity of *only* positions 7, 13, and 14 in isolation (i.e., disregarding the other 17 positions). 3-position subsets are used in this work because they allow for a unique 3-dimensional lrmsd superposition and are more computationally tractable than subsets of size 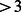, while still allowing for binding site position decomposition.

The dissimilarity between a pair of substructures is quantified by a combination of structural distance and chemical feature dissimilarity introduced in [26]. Specifically, the distance between any two substructures 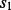 and 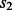 is expressed as:
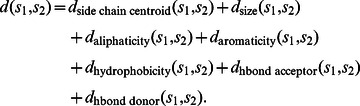
The 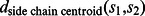 term is the pairwise-aligned side chain centroid lrmsd between the substructures. The remaining terms account for differences in the amino acid properties between the substructures 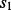 and 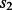 as quantified by the pharmacophore feature dissimilarity matrix as defined in [26].

For a given set of residue positions, we can calculate a matrix of pairwise distances between substructures using the distance measure defined above. Each row can be thought of as a feature vector that represents how different a protein is with respect to all other proteins in terms of the selected residues. The distance matrix is highly redundant. We use Principal Component Analysis (pca) to obtain a low-dimensional embedding. Our previous work [27] showed that this dimensionality reduction typically results in negligible information loss. Some technical details on how we correct for overrepresentation are described in *Text S3*. The dimensionality-reduced feature vectors are then clustered to identify sub-groups that share strong structural similarity. The number of clusters is not known beforehand and the number of clusters will vary depending on the set of positions being compared. The Gaussian Mixture Model (gmm) clustering method implemented in the mclust package [28] was used to identify both the number of clusters present and the cluster memberships for each of the feature vectors.

### Selecting Highly-Predictive Clusters (HPCs)

The above feature vector computation and clustering steps are repeated for each possible 3-position subset in order to compare *all* possible local structural features across *all* proteins. Structural variation in most subsets is not expected to be informative, either because no significant variation is present, or because spurious patterns can occur due to chance. However, functionally relevant structural variation can be detected with many different subsets and therefore distinguished from random patterns, as will be shown below.

**Figure 1:**
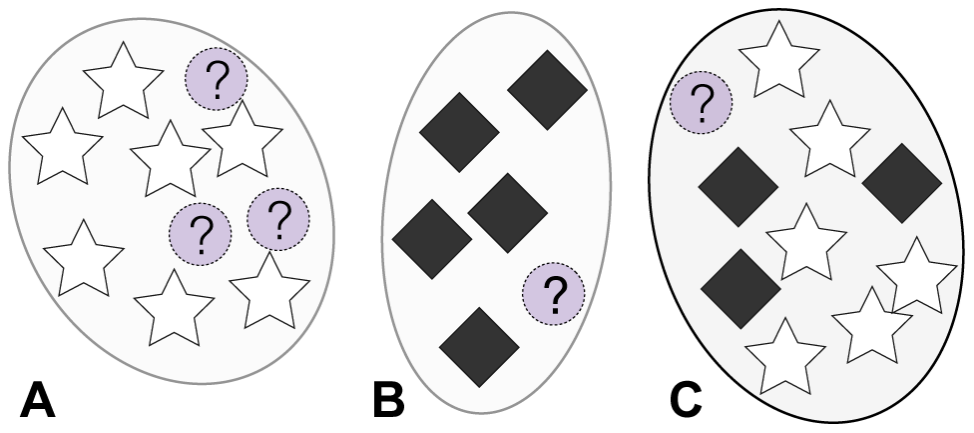
Illustration of cluster evaluation procedure. The star and diamond symbols represent structures with known labels and the question marks represent structures with an unknown label. Clusters A and B will both be selected as HPCs for their respective labels (star and diamond, respectively) because they are each pure in a single label (unknown labels are disregarded). Cluster C will not be selected as an hpc because it has low purity.

A cluster that is dominated by one annotation label can be used to predict the label for other structures in that cluster whose annotation is unknown. We therefore call such clusters “highly predictive” (HPCs). Identification of HPCs is performed by selecting a minimum threshold for the label purity of clusters, and then selecting all clusters with equal or greater label purity than this minimum as HPCs; we used the strictest purity threshold possible (1.0 or 100% purity) in this work (see Fig. 1). In general, purity is calculated for a multiset of labels, 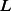, as 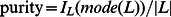 where 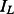 is the multiplicity function of a label within the multiset 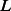 and 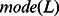 is the most frequent label within 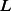. As with the dimensionality reduction mentioned above, we need to correct for overrepresentation bias, the details of which are described in *Text S3*. Purity alone does not account for the distinctness of the proteins in the cluster relative to the remainder of the dataset. For example, an hpc for label 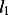 that partially overlaps a second hpc for label 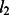 is less likely to be informative than an 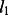 cluster greatly separated from the remainder of the dataset. The “degree of separation” or “distinctness” of a cluster was quantified by calculating the cluster silhouette score [29]. The mean silhouette score for a cluster was then used as a further selection criteria for identifying HPCs by removing potential HPCs with negative average silhouette scores (malformed clusters).

### Support Vector Machine-based (SVM) decision boundary

Each time an unlabeled protein falls within an hpc, that protein receives a single vote in favor of the majority label associated with the hpc. Because a protein can be a member of at most one cluster per 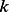-position subset, the maximum number of votes any protein can receive is equal to the number of possible 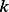-position subsets. For any given 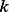-position subset, it is possible that all clusters are HPCs or that no clusters are HPCs, depending on how the labels are distributed among the clusters. It is also possible that a protein may never fall within any hpc and therefore would receive zero votes for any label; such proteins are excluded from further analysis after the voting step. In the experiments described below this case rarely occurred. After tallying the label votes across all 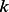-position subsets, the label predicted for a given structure is determined by an SVM-derived decision boundary as described below.

Given a set of label votes that have been determined for an unlabeled structure, the threshold(s) used to decide which of the two or more label classes to assign to the structure requires the definition of a decision boundary procedure. For example, given a set of annotation labels containing the label classes 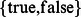 (e.g., indicating whether a kinase is known to bind to a given ligand), a simple decision rule may be that given a structure with 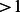 true vote, predict the true label for that structure. However, determining a single threshold for deciding the number of label votes required to classify a structure into one of several classes is difficult to generalize.

**Figure 2:**
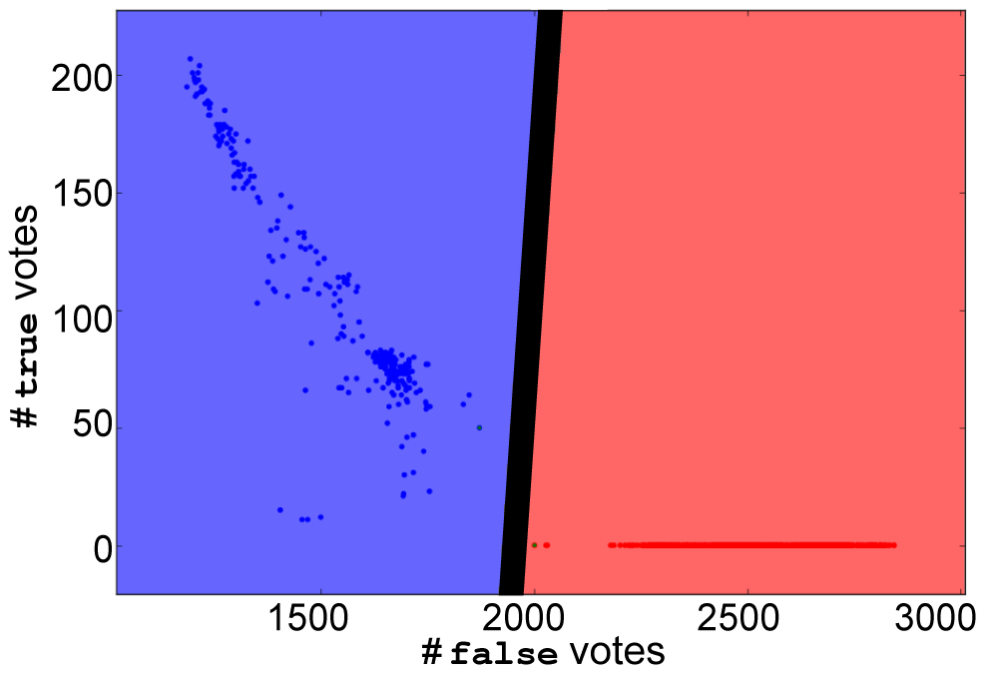
Decision boundary for label vote vectors computed by SVM. In the above scatter plot, each point corresponds to the number of true/false votes accumulated by each substructure across all clusterings. Combining the above label vote vectors with the known labels for substructures to train an svm (using linear kernel) results in the decision boundary shown as the bold black line. The red and blue regions (right and left sides of the boundary, respectively) denote the values for which the predicted label will be **false** and **true**, respectively. Blue points indicate substructures known to have the **true** label while red points denote the **false** label. In the case of Roscovitine above, wide separation between the two classes exists.

Because ccorps is a semi-supervised approach, the labels for the training structures are known and can be used to empirically estimate a vote count decision boundary. For example, given structure 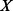 with known label, the number of times that 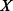 appeared in a **false** hpc or a **true** hpc, across all 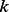-position subsets, can be calculated using the same approach as for unlabeled structures. The structure 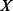 is then represented by an 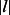-dimensional vote vector, where each of the 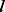 dimensions corresponds to the number of votes 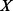 received for label 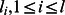 (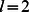 for the case of kinase binding affinity, since we only have false and true labels). Application of this procedure to all labeled structures in the dataset provides an empirical basis for calculating a decision boundary in the vote space given the vote distribution for labeled structures. For example, the blue and red points shown in the scatter plot of Fig. 2 denote the vote vectors for training set substructures with known **true** and **false** labels, respectively.

Given the vote vectors calculated for all labeled training set substructures in the dataset, it is then possible to train any number of classifiers in order to determine a decision boundary. To compute a decision boundary in the vote space for classifying unlabeled proteins, svms were selected in this paper. First, an svm (linear kernel) is trained using the vote vectors of labeled training set substructures. For example, the decision boundary determined by training an svm on vote vectors is shown in Fig. 2 as the bold, black line. Next, for an unlabeled substructure with a given vote vector, the label for the substructure can be predicted by determining which side of the svm decision hyperplane the unlabeled structure falls within. As illustrated in Fig. 2, test vote vectors falling within the blue region will be predicted as having the true label and those falling within the red region, the **false** label. For training svms and calculating the 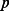-values of predictions made by those svms, libSVM [30] was used in this work.

### Validation experiments and method generalization

To validate the predictive ability of the structural features identified by ccorps an extensive dataset of 48 families was automatically constructed using the Pfam database [17] as a source of well-curated protein alignments. The annotation labels analyzed in the validation set were per-structure Enzyme Commission (EC) number classifications. Cross-validation was performed in order to evaluate the predictive power of ccorps and the utility of the distinguishing structural features identified. The overall classification accuracy of ccorps (*Text S2*) when applied to the validation dataset demonstrates the ability of ccorps to identify structural features that distinguish functionally different protein homologs and the ability of ccorps to generalize to non-kinase protein families.

## Results

First, we will introduce the components of the kinome structure and affinity datasets used in this work. Next, structural features of the kinase binding site that are identified by ccorps to be predictive of inhibitor binding ability are presented. Then, cases of these predictive structural features that are common to phylogenetically diverse sets of kinases are highlighted. Finally, the performance of ccorps for predicting the binding ability of inhibitors across the kinome is quantified and compared to the related approach of Jackson et al. [9] as well as a sequence-based approach.

### Dataset

In order to enable the kinome-scale analysis of the protein kinase atp binding site presented here, a dataset of protein kinase binding site structures was assembled and then mapped to the affinity dataset of Karaman et al. [31]. Karaman et al. studied the affinity of 38 kinase inhibitors across 317 kinases and was one of the most comprehensive studies of kinase inhibitor selectivity at that time. Mapping a structure-affinity-phylogeny dataset by further incorporating the kinome family labeling of Manning et al. [2] has enabled the incorporation of all available crystallographic structures of the atp binding site and the analysis of shared structural features between major kinase families that is presented later in this paper.

#### Kinase structure dataset

The kinome structural dataset was constructed from all structures (domains) annotated as belonging to pfam:Pkinase and pfam:Pkinase_Tyr (all e pk domains, apks excluded) in release 25 of pfam (2154 total domains before filtering). After the binding site residue positions to analyze were selected, as detailed in the following section, and proteins having one or more gaps at those positions were excluded, a total of 1958 structures remained. These 1958 structures correspond to 208 unique kinase proteins. The distribution of sequences and structures across the seven major kinome families is shown in Table 1. Of the 1958 kinase structures within the dataset, 1281 (65.4%) were part of the kinome inhibitor affinity dataset of Karaman et al. [31] and therefore have known annotation labels for each of the 38 inhibitors that were experimentally determined by Karaman et al. [31]. The dataset contained a large number of active DFG-in, inactive DFG-out and other conformations.

**Table 1:** Statistics for the human kinome dataset.

Kinase Family	AGC	CAMK	CK1	CMGC	Other	STE	TK	TKL	Unclassified
# Structures	171	231	20	500	114	55	445	58	364
# Sequences	19	34	6	33	18	17	47	9	75
# Annotated	6	13	2	16	5	11	35	6	43

#### Binding site position selection

**Figure 3:**
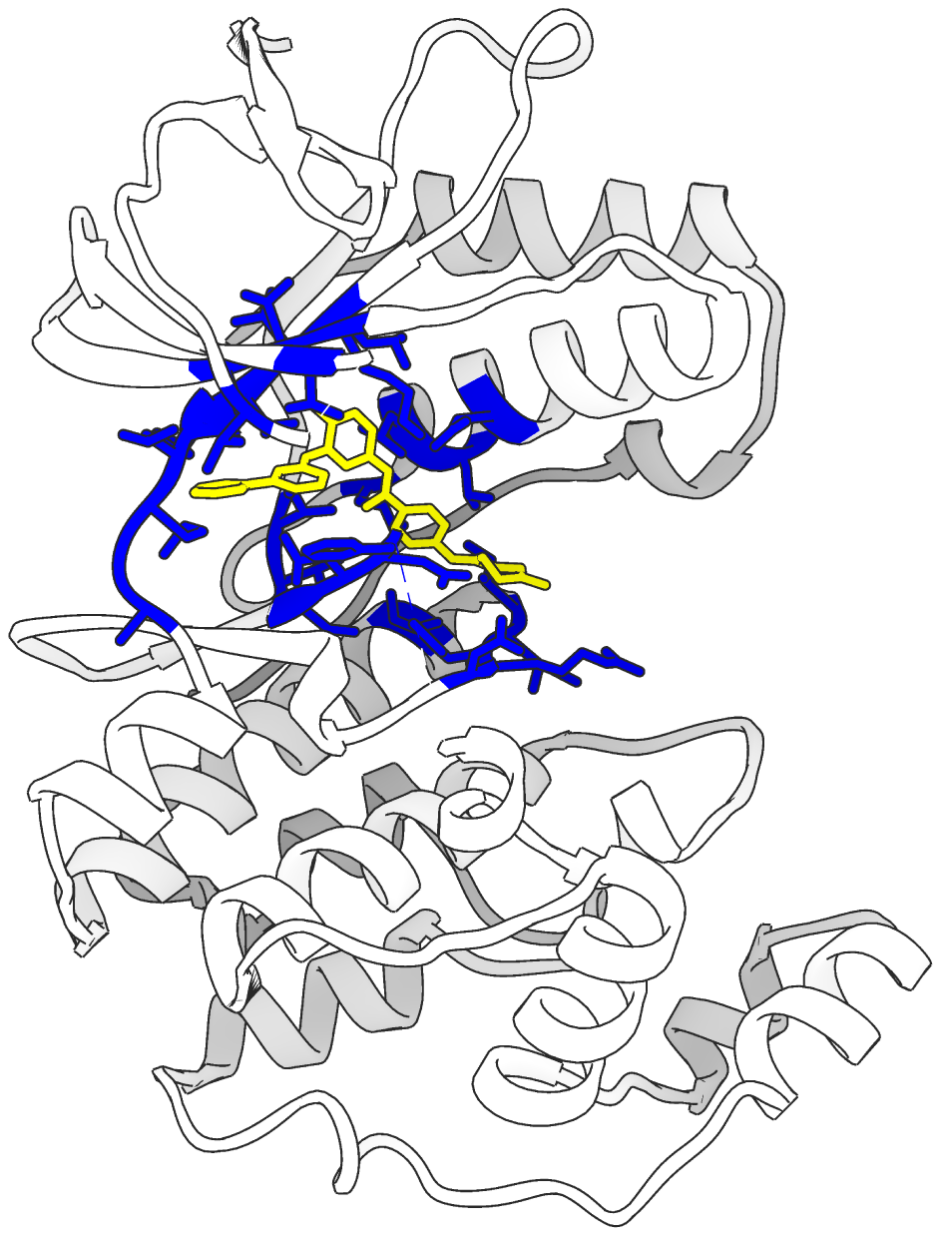
Kinase binding site definition: The 27 alignable residue positions (blue) within 5 Å of the bound imatinib molecule (yellow) are mapped on to protein kinase structure pdb:3hec.

All residues having one or more atoms within 5 Å of one or more imatinib atoms from the imatinib-bound structures pdb:2pl0 or pdb:3hec were selected as candidate binding site positions. Candidate positions were eliminated if they corresponded to highly gapped columns in either the pfam:Pkinase or pfam:Pkinase_Tyr Multiple Sequence Alignments (msas). After filtering, 27 binding site residue positions remain (shown in Fig. 3): 30, 38, 51–53, 71, 74, 75, 78, 83, 84, 104–109, 111, 146–149, 157, 166–169 (residue numbering according to pdb:3hec). Imatinib was chosen as a reference inhibitor for selecting the binding site positions to include in the analysis because it is of the large type II kinase inhibitor variety and extends both into the atp binding pocket as well as the neighboring allosteric pocket. The cutoff distance of 5 Å was selected in order to be consistent with the binding site selection cutoff chosen by Jackson et al. [9]. For full details on the mapping and alignment of positions among structures within the kinase dataset please refer to *Text S1*.

#### Kinase inhibitor affinity dataset

The affinity (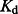) for 38 small molecule kinase inhibitor compounds was determined for a set of 317 kinases using an *in vitro* competition binding assay by Karaman et al. [31]. The 38 inhibitors tested include staurosporine, 1 lipid kinase inhibitor, 15 serine-threonine kinase inhibitors and 21 tyrosine kinase inhibitors. Affinity values were mapped from the Karaman et al. [31] dataset to the kinome structural dataset by mapping the ncbi RefSeq IDs provided by Karaman et al. [31] to the UniProtKB IDs [32] of the proteins in the structural dataset. 137 of the 208 protein sequences in the structural dataset mapped to the affinity dataset published by Karaman et al. [31].

In order to simplify the problem of correlating structural features with binding affinities, the binding affinity (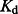) values were binned into 2 classes (**true**/**false**) by thresholding the affinity values at 10 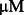 (i.e., 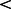
10 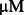 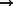 **true**; 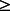
10 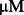 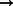 **false**). This 10 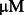 cutoff between the two label classes was used consistently across all inhibitors and selected because it is the largest 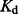 considered by Karaman et al. [31] to be meaningful for inhibitor binding in the screening dataset used in their work [31].

### Interpretation of Highly Predictive Clusters (HPCs)

**Figure 4:**
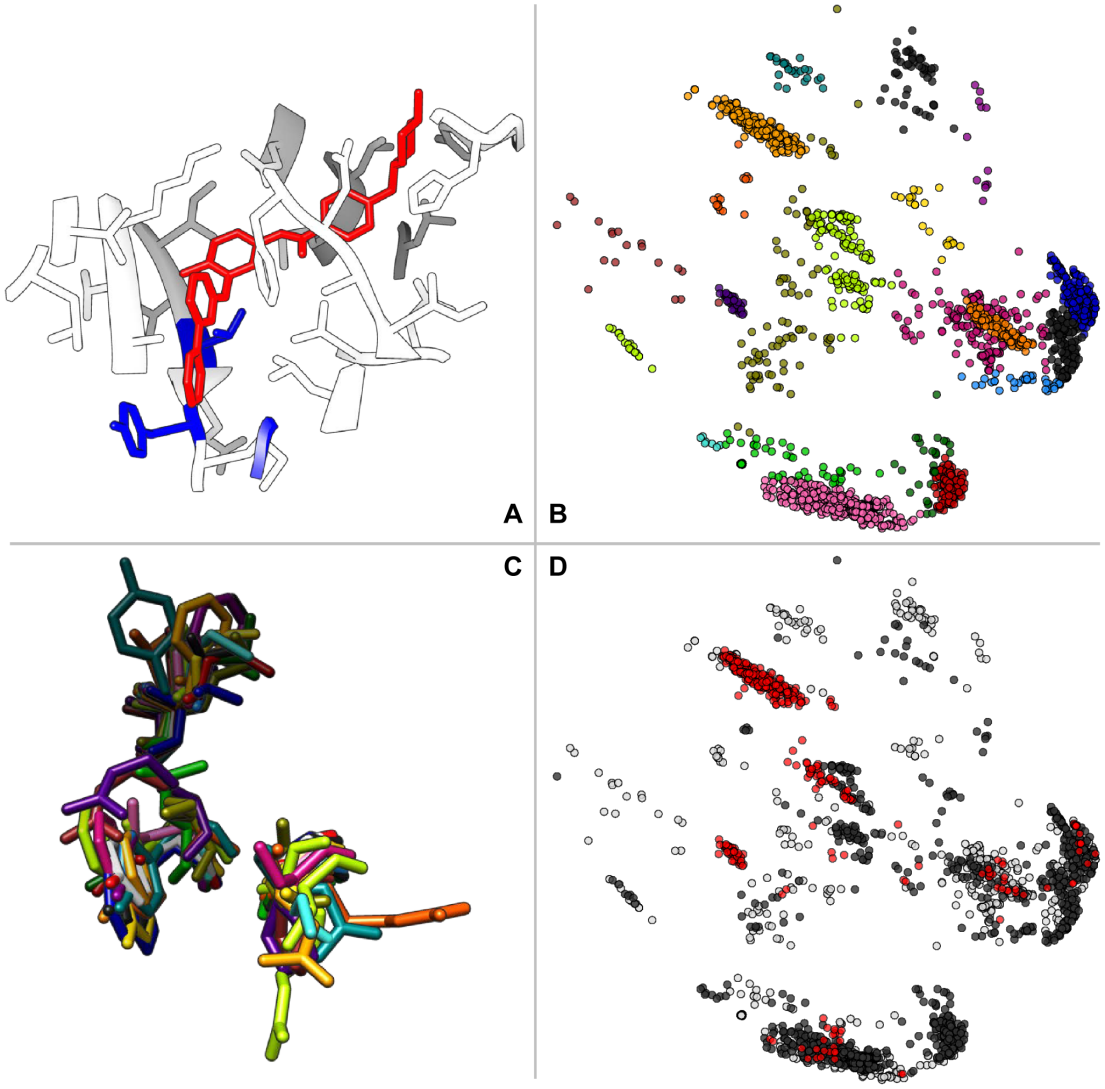
Highly predictive clusters. (A) Structure of lck (PDB:2pl0) with a 3-position substructure shown in blue stick representation (Thr-316, Tyr-318, Gly-322) and bound imatinib molecule in red. (B) Substructure embedding computed by ccorps when comparing the 3-positions shown in A across the entire 1958 structure dataset. Each point in the clustering represents a single 3-residue substructure. The coloring indicates the cluster membership of each substructure (21 clusters in total are shown). (C) Aligned 3-residue substructure representatives, from each of the 21 clusters identified by ccorps, for the 3-position subset shown in A. The color of each substructure corresponds to its cluster assignment. (D) Same embedding as in B, but now colored according to affinity. The red and black coloring of each point indicates **true** and **false** affinity labels for flavopiridol, respectively, while white indicates substructures lacking affinity annotations.

The process by which ccorps recognizes structural features that are associated with kinase binding affinity is through the identification of Highly Predictive Clusters (HPCs). Given the 27-position binding site (Fig. 3), ccorps computes a clustering for each of the 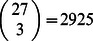 unique 3-position subsets. For example, consider the 3-residue substructure shown in Fig. 4A. The 3 residues shown correspond to 3 positions in the full kinome alignment and the corresponding residues for each structure in the kinome dataset are structurally compared to compute the substructure clustering shown in Fig. 4B. Each of the 1958 substructures within the kinase structure dataset is shown in Fig. 4B as a single point. The color of each point in Fig. 4B corresponds to the cluster assignment as computed by ccorps.

Several informative observations regarding kinase structural diversity and its association to inhibitor binding affinity can be made by further examination of the substructure clustering shown in Fig. 4B. Immediately upon examination of the substructure clustering it can be noted that multiple distinct clusters of kinases exist. This observation alone indicates that the 3-position substructure that resulted in this clustering is highly diverse among kinase binding sites. Conversely, the presence of a single large cluster would indicate that the 3-position substructure was structurally conserved, exhibiting little variance across the kinome; indeed instances of clusterings with a single dominating cluster were also observed for some 3-position subsets. As demonstrated in Fig. 4C, where one randomly selected representative substructure is shown for each of the 21 clusters identified by ccorps, both the geometry and residue types vary significantly for this 3-position subset.

By incorporating the affinity annotation labels for a particular inhibitor, further observations can be made about the association between the 3-position substructure shown in Fig. 4A and the kinases capable of binding that inhibitor. For example, mapping the affinity annotation labels for the inhibitor flavopiridol onto the substructure clustering (Fig. 4D) reveals that some of the clusters consist of only a single annotation label while others are a mixture of labels. In Fig. 4D, kinases capable of binding flavopiridol are colored red (**true** label), kinases incapable of binding flavopiridol are colored black (**false** label) and kinases lacking affinity annotation are colored white (undefined label). As shown in Fig. 4D, multiple clusters of purely **true** labels exist and are considered to be HPCs by ccorps.

The existence of true-only clusters indicates that the 3-positions shown in Fig. 4A are a distinguishing structural feature for identifying kinases that bind flavopiridol. More interestingly, however, is the fact that multiple, structurally distinct versions of the same 3-position substructure exist for different kinases that all are capable of binding flavopiridol. This result is significant because it indicates that across the kinome there are different structural motifs that are associated with binding flavopiridol, as opposed to a single, shared structural motif across all flavopiridol-binding kinases. The ability to identify multiple structural motifs that can each be associated with inhibitor binding is a strength of ccorps.

Furthermore, the existence of clusters containing only kinases *incapable* of binding flavopiridol can also be observed in Fig. 4D. These HPCs are also informative because they identify particular structural versions of the 3-position substructure in Fig. 4A that are all incapable of binding flavopiridol. Finally, clusters consisting of a mixture of kinases that are both capable and incapable of binding flavopiridol can be identified in Fig. 4D. For kinases in these clusters, the 3-position substructure is not a distinguishing feature of flavopiridol-binding ability.

**Figure 5:**
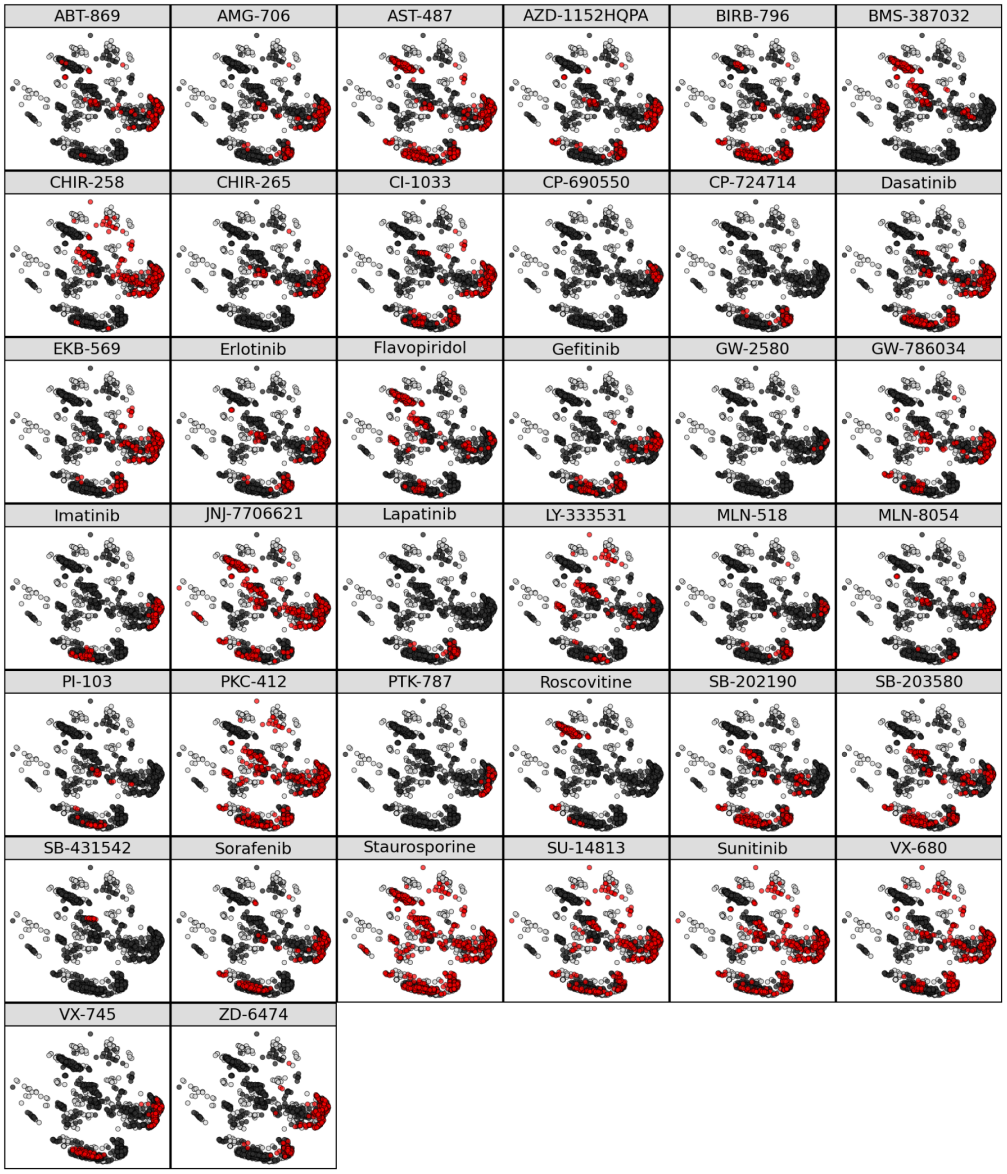
Affinity annotation labeling for all 38 inhibitors. The substructure clustering computed for the same 3 positions examined in Fig. 4 is relabeled above for each of the 38 inhibitors included in the dataset. In each cell above, red and black indicate the **true** and **false** affinity labels, respectively, for each inhibitor, while white indicates a lack of annotation. As can be noted by comparing the distribution of red points across the different inhibitors, for most inhibitors, the kinase proteins capable of binding to them are not distributed in a single cluster, indicating structurally diverse features exist among the kinases selected by each inhibitor.

Finally, while flavopiridol is discussed in detail here for illustration, the same analysis was computed by ccorps for each of the 38 different inhibitors within the affinity dataset. For each of the inhibitors, the affinity labels can be mapped separately onto the same substructure clustering as shown in Fig. 5. However, it should be noted that no information is shared between the results for different inhibitors in this work; that is, each inhibitor is computed in a fully separate ccorps computation (the substructure clusterings do not vary, just the annotation labels).

Examination of the affinity-annotated substructure clusterings shown in Fig. 5 reveals that the set of clusters which are HPCs varies greatly depending on the inhibitor considered. While the flavopiridol-annotated substructure clustering contains multiple HPCs for both **true** and **false** labels, the correspondingly annotated clustering for other inhibitors, such as VX-745, PI-103 and imatinib, contain only **false** HPCs. This result demonstrates that the substructures that are informative of inhibitor binding are inherently inhibitor-specific. That is, a subset of binding site positions that are predictive for one inhibitor are not necessarily predictive for another inhibitor.

It is important to note that Fig. 4 and Fig. 5 represent the same clustering for just *one* 3-residue substructure. However, *all* 2925 clusterings are computed and *all* HPCs detected in these clusterings are used to predict binding affinity. The particular three-residue subset shown in Fig. 4A was chosen because the resulting clustering exhibits a number of illustrative features. First, the clustering is relative “clean” with well-separated clusters. Second, it contains highly predictive clusters for both binding and not binding to flavopiridol (the ocher cluster in the top-left and the red cluster in the bottom right of figure Fig. 4B, respectively). None of these features are essential for predicting binding affinity; all automatically selected HPCs in all clusterings are used to predict affinity, each casting one “vote.”

### Phylogenetically diverse HPCs

Numerous instances of cross-family affinity for both type I and II kinase inhibitors have been identified, as was clearly illustrated by the kinome affinity maps created by Karaman et al. [31]. It is important to identify structural features shared among phylogenetically diverse kinases that share affinity for a particular inhibitor, because they provide a basis for reasoning about inhibitor cross-reactivity when overall sequence similarity will be low. Furthermore, by identifying these shared structural features, it may be possible to rationally re-engineer the specificity of inhibitors by avoiding the targeting of these features, since they are not unique to the intended kinase target. In order to identify the number of instances of cross-family structural features that can be associated with specific inhibitor binding, the distribution of substructure clusters across all 3-position subsets was analyzed.

**Figure 6:**
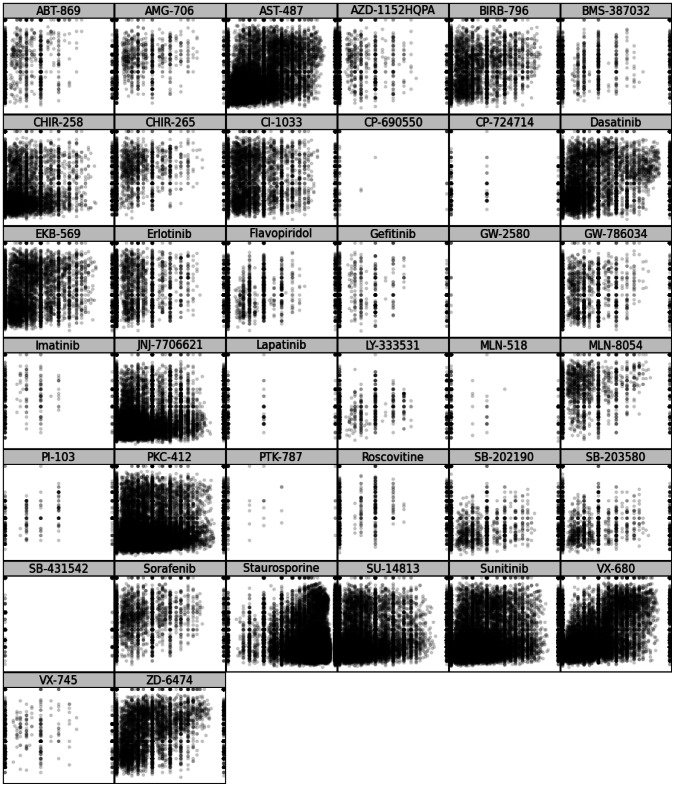
Distribution of phylogenetic and affinity purity cluster scores for all 38 inhibitors. As can be seen in the case of drugs such as imatinib and lapatinib, very few clusters that have a majority of **true** labels were identified, yet clusters of phylogenetically diverse structures all having **true** labels can be identified. Staurosporine exhibits a reflected distribution relative to the other drugs, because due the nature of its non-selectivity across the kinome, instances of phylogenetically distant structures that exhibit Staurosporine affinity are common. Refer to Fig. 7 for additional details.

**Figure 7:**
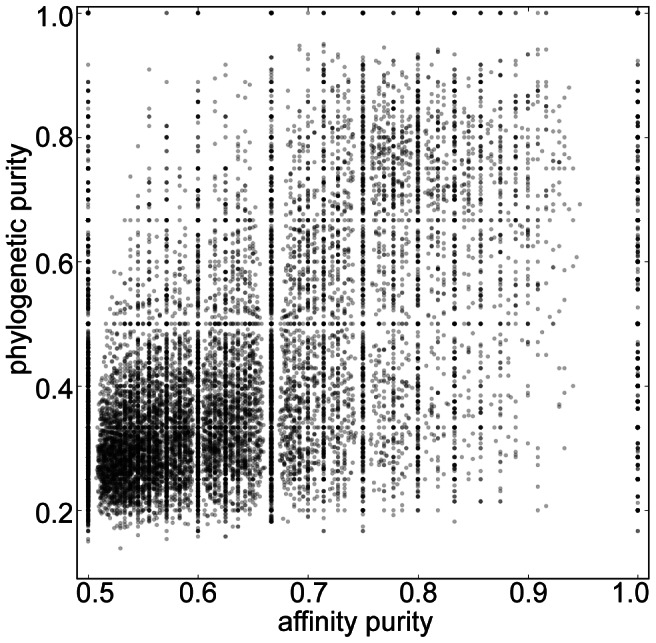
Distribution of phylogenetic and affinity purity cluster scores for VX-680. Each point in the scatter plot above marks the purity for the drug affinity **true** label on the 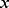-axis and the phylogenetic label purity on the 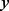-axis. For example, a point above located at the coordinates 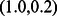 denotes a cluster that is 100% pure in the **true** drug affinity label (for VX-680 in this case) but is only 20% pure in the most common phylogenetic label present; that is, this cluster indicates one instance of structural similarity among phylogenetically diverse proteins that also coincides with having affinity for VX-680. Conversely, a point at the coordinates 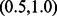 indicates a cluster that contains only structures from one phylogenetic (family-level) branch but contains an equal proportion of **true** and **false** affinity labels; that is, a case where structurally similar, closely related (phylogenetically) structures have different affinities for VX-680. Each point is semi-transparent so that darker areas in the plot indicate a higher density of points.

Each individual cluster, across all 2925 clusterings and all 38 inhibitors, was evaluated to calculate the purity of both affinity labels and family-level phylogenetic labels. For example, a cluster containing 3 distinct kinase sequences with affinity labels 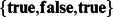 and family labels {agc, camk, tk} would have an affinity purity of 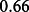 and a phylogenetic purity of 0.33. By plotting the affinity and phylogenetic purity scores of each cluster (separately for each inhibitor) as shown in Fig. 6, the distribution of clusters across the spectrum of possible scores can be evaluated. Note that only the clusters having a true label majority are plotted in Fig. 7 (i.e., a true label majority is 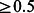 purity in the true label). Additionally, Table 2 lists per inhibitor statistics for cluster distributions shown in Fig. 7.

**Table 2:** Phylogenetically diverse HPC statistics per inhibitor.

Inhibitor	# true -HPCs	# 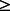 2 Families
ABT-869	345	249
AMG-706	274	202
AST-487	2415	1955
AZD-1152HQPA	506	374
BIRB-796	893	730
BMS-387032	728	447
CHIR-258	1577	800
CHIR-265	242	184
CI-1033	1247	704
CP-690550	11	5
CP-724714	115	89
Dasatinib	1848	1193
EKB-569	1133	684
Erlotinib	596	456
Flavopiridol	921	481
GW-2580	0	0
GW-786034	1443	809
Gefitinib	203	169
Imatinib	57	45
JNJ-7706621	4087	2761
LY-333531	634	314
Lapatinib	115	89
MLN-518	92	70
MLN-8054	435	301
PI-103	182	69
PKC-412	3419	2368
PTK-787	7	6
Roscovitine	593	335
SB-202190	644	513
SB-203580	738	546
SB-431542	133	69
SU-14813	4415	3116
Sorafenib	561	405
Staurosporine	17098	14802
Sunitinib	5525	4077
VX-680	2707	1786
VX-745	189	151
ZD-6474	1059	610

For each inhibitor, the total number of true -HPCs (column “# true -HPCs”) is shown. The subset of true -HPCs that consist of proteins from two or more of the kinase families defined by Manning et al. [2] (column “# 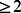 families”) are also shown. The multitude of true -HPCs that include proteins from distinct families of the kinome can be noted by the relatively large percentage (73% overall across all inhibitors) of HPCs that span families. All of these 41964 instances of structurally similar features between families are provided in *Dataset S1*.

In order to build intuition for interpreting the cluster distributions, the cluster distribution for VX-680 (Fig. 7) is examined in more detail because it is representative of the distribution for many of the other inhibitors. As listed in Table 2, 23,495 clusters were identified by ccorps that have 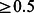 purity in the true label for VX-680 (hereafter referred to as **true**-majority clusters). Only these **true**-majority clusters are plotted in the cluster distribution shown in Fig. 7, meaning the minimum “affinity purity” displayed in Fig. 7 is 0.5 by definition (because only 2 different affinity labels exist, **true** and **false**).

As can be seen in Fig. 7, the vast majority of clusters identified by ccorps have low affinity purity as well as low phylogenetic purity. This is to be expected because highly conserved portions of the kinase atp binding site are known to exist. Structural features that consist of conserved residue positions will be common to many kinases from different families due to the fact that these positions are so heavily conserved, which explains the low phylogenetic purity of these clusters. Furthermore, these conserved features are unlikely to be correlated with the affinity for a particular inhibitor because most inhibitors have been engineered to not have broad cross-reactivity across the kinome. Staurosporine is an exception as it is a very non-selective inhibitor due to its interaction with highly conserved binding site features; the cluster distribution corresponding to staurosporine (Fig. 6) is markedly different from the other inhibitors with most clusters having high affinity purity across a range of phylogenetic purity values.

Examination of the extremes of the VX-680 cluster distribution reveals further insights into the frequency of structural similar features among kinases with different degrees of sequence similarity. Clusters having a phylogenetic purity of 1.0 (i.e., all proteins belong to the same family) but having low affinity purity exist, and for VX-680 276 such clusters were identified by ccorps. This observation is interesting because it illustrates that kinases sharing sequence similarity (relative to kinases outside the family) have multiple common structural features that are not informative of the ability of these kinases to bind VX-680 and are therefore unlikely to be good features to target in design studies. Because ccorps only incorporates clusters with high affinity purity (i.e., HPCs), these conserved structural features that are not indicative of VX-680 binding are ignored by ccorps when predicting affinity for unannotated kinases. This observation can also be made for each of the other inhibitors as shown in Fig. 6.

Another interesting extreme of the VX-680 cluster distribution to examine is the existence of HPCs that are phylogenetically diverse. The HPCs selected by ccorps correspond to the right-most column of points in Fig. 7; these clusters all have an affinity purity of 1.0 for VX-680 and therefore contain *only* structures with known VX-680 affinity. As can be noted in Fig. 7, HPCs exist at a range of phylogenetic purity values. ccorps identified a total of 2707 HPCs for VX-680, and 1786 (66%) of these HPCs contain proteins belonging to two or more distinct kinase families. This result demonstrates that ccorps is capable of identifying cross-family structural features that are associated with VX-680 binding. Furthermore, this result is not unique to VX-680. As shown in Fig. 6 and tabulated in Table 2, cross-family structural features associated with inhibitor binding were identified for all of the inhibitors tested with the exception of GW-2580, for which no true-majority clusters were identified.

Examination of the cluster distributions across each of the inhibitors reveals a wide range of observations. While many inhibitors have a cluster distribution similar to that of VX-680, for some inhibitors ccorps identified relatively fewer true-majority clusters. For example, only 133 clusters with affinity purity 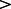
0.5 were identified by ccorps for SB-431542 and all of these happen to be HPCs. However, even among this relatively low number of HPCs, 69 (52%) of the clusters contain kinases from two or more families. As demonstrated by the corresponding distributions for all 38 inhibitors in Fig. 6, such shared structural similarity is not rare.

### Predicting kinase-inhibitor binding

The approach used by ccorps to classify an unlabeled kinase is to identify the cluster to which the unlabeled kinase belongs. If the associated cluster is an hpc, the label for the hpc is transferred to the unlabeled kinase. Non-informative clusters containing a mix of labels (non-HPCs) do not contribute to the label prediction process. This “co-clustering” analysis approach is repeated for all of the 2925 substructure clusterings and the final label prediction for an unlabeled kinase is then selected as detailed in *Methods*.

The ability of ccorps to predict the binding of each inhibitor for proteins within the annotated structural dataset was assessed using the cross-fold validation approach described in the following section. For each of the 38 inhibitor annotation label sets, an independent evaluation of ccorps was performed. No information was shared among the evaluations in order to validate the predictive ability of ccorps to identify structural features predictive of the binding ability of each inhibitor independently.

#### Cross-fold validation

To assess the utility of HPCs for identifying substructure positions indicative of functional specialization, cross-fold validation was performed for each family within our dataset. The structures within a protein family were first divided into 70% sequence identity groups (nr-clusters) so that no protein in a test set shares 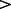
70% sequence identity with any protein in the training set. The sequence identity is computed over the domain (i.e., not the whole sequence nor just the binding site). Because of the non-uniform distribution of structures across the nr-clusters, the number of structures in the test set varies with each fold. In each fold, structures that were part of the test set are marked with label unknown, and are disregarded when calculating the purity of clusters (defined in *Methods*) during the hpc selection step, just as the structures with truly unknown label. Finally, standard 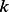-fold cross validation was performed with each of the nr-clusters each being one fold (i.e., 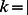 nr-clusters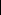). Given the nr-clusters-based fold partitioning above, the training set is used to identify HPCs and train an svm-based classifier to predict labels for kinases in the test set.

#### Prediction performance

**Figure 8:**
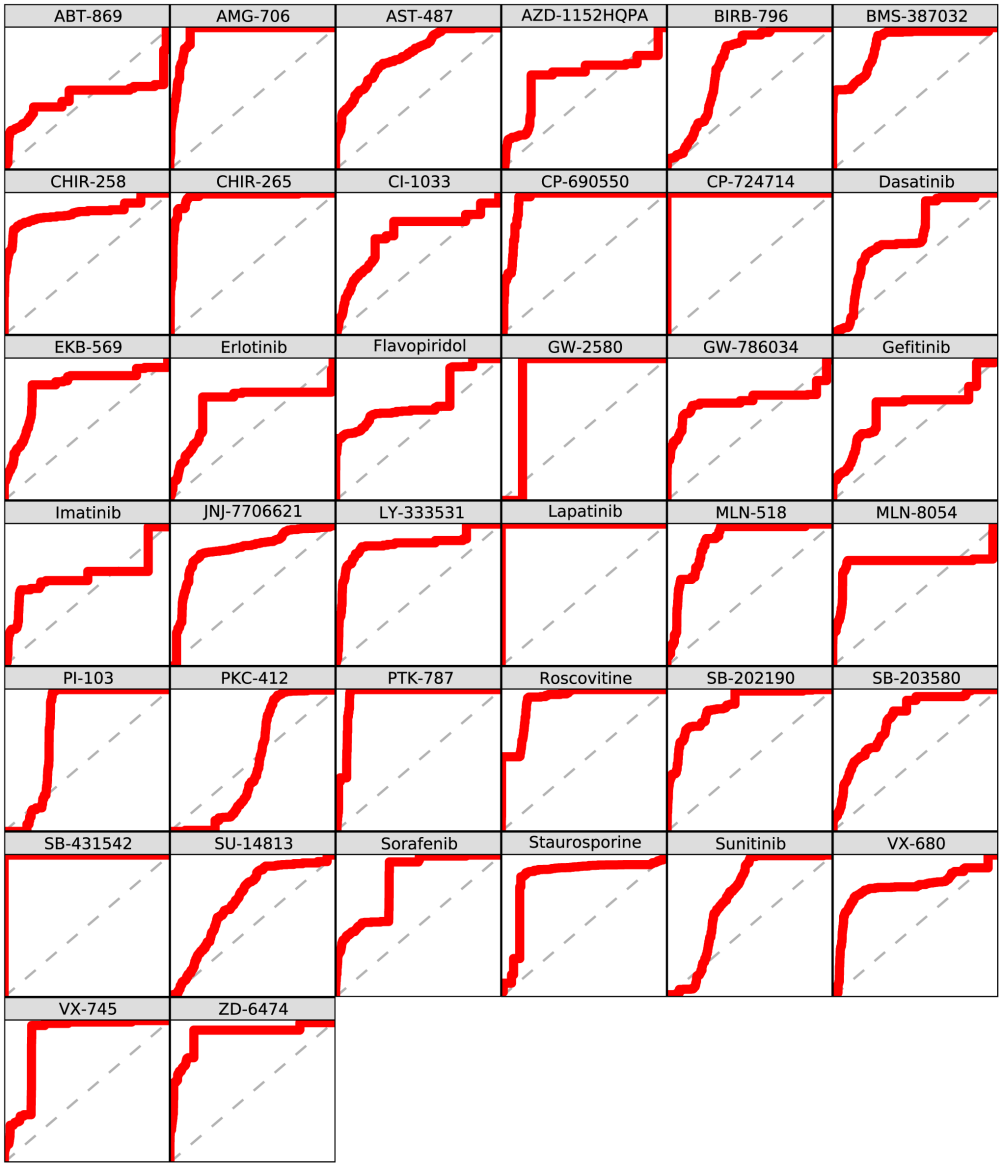
Per inhibitor Receiver Operator Characteristic (ROC) curves. The 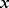
- and 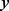-axis plot (1-specificity) and sensitivity, respectively, both ranging from 0 to 1. The Area Under Curve (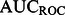) as well as the 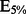 per drug can be found in Table 3. As shown above, ccorps is able to construct a near-perfect classifier for several drugs, such as PI-103, SB-431542. The classifiers constructed for some inhibitors, such as flavopiridol, are able to achieve high precision, but only at low sensitivities (recalls), as further illustrated by the pr curves in Fig. 9.

**Figure 9:**
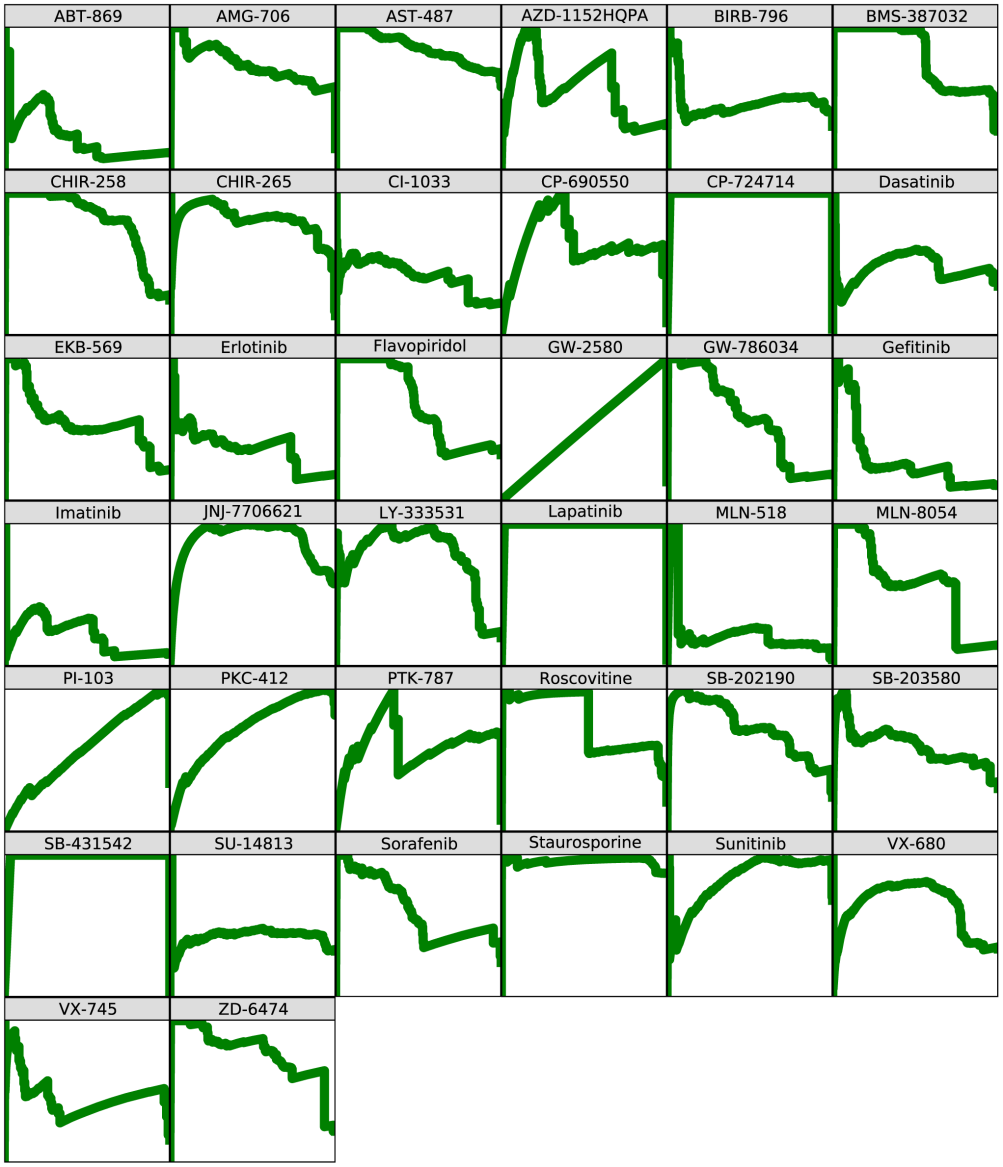
Per inhibitor Precision-Recall (PR) curves. The 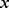
- and 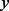-axis plot the recall and precision, respectively, both ranging from 0 to 1. The Area Under Curve (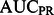) per drug can be found in Table 3. As shown above, ccorps is demonstrated to have very high precision across a wide range of inhibitors when tested for targets spanning the kinome.

For each of the 38 inhibitors included in the affinity dataset, ccorps was used to predict the set of kinases able to bind to that inhibitor. The performance of ccorps was assessed for each inhibitor, independently, by computing the Receiver Operator Characteristic (roc) curve for the set of predictions, which evaluates the sensitivity (# true positives/(# true positives + # false negatives)) at each specificity (# true negatives/(# true negatives + # false positives)) value. The roc curves for the predictor constructed by ccorps are shown in Fig. 8 for each inhibitor and the Area Under Curve (auc) for each roc curve is listed in Table 3. Additionally, the Precision-Recall (pr) curve for each inhibitor can be found in Fig. 9. The pr curve plots the precision (# true positives/(# true positives + # false positives)) versus the recall (equivalent to sensitivity).

**Table 3:** Affinity prediction performance of ccorps for the kinase inhibitors.

	CCORPS			Jackson et al. [9]	Sequence-based		
Inhibitor	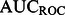	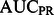	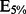 / 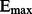	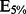 / 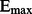	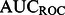	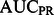	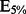 / 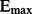
ABT-869	0.50	0.23	0.51 (4.27/8.41)		0.64	0.36	0.59 (5.00/8.43)
AMG-706	0.96	0.74	0.87 (5.91/6.77)		0.77	0.56	0.84 (5.66/6.71)
AST-487	0.81	0.86	1.00 (1.71/1.71)		0.84	0.90	1.00 (1.71/1.71)
AZD-1152HQPA	0.65	0.27	0.46 (3.12/6.77)		0.69	0.34	0.45 (3.07/6.78)
BIRB-796	0.75	0.48	0.51 (1.67/3.28)	0.91 (3.65/3.98)	0.54	0.33	0.16 (0.51/3.27)
BMS-387032	0.88	0.80	1.00 (3.69/3.69)		0.93	0.88	1.00 (3.69/3.69)
CHIR-258	0.86	0.81	1.00 (4.05/4.05)		0.93	0.85	1.00 (4.05/4.05)
CHIR-265	0.97	0.81	0.90 (6.61/7.31)		0.91	0.71	0.67 (4.86/7.24)
CI-1033	0.70	0.42	0.56 (2.48/4.47)		0.77	0.57	0.94 (4.20/4.48)
CP-690550	0.94	0.22	0.25 (8.33/32.79)		0.35	0.03	0.05 (1.54/32.85)
CP-724714	1.00	0.99	0.86 (20.30/23.69)		0.71	0.07	0.00 (0.00/23.72)
Dasatinib	0.70	0.48	0.27 (0.78/2.90)		0.74	0.67	0.94 (2.71/2.89)
EKB-569	0.79	0.56	0.71 (3.31/4.63)		0.82	0.60	0.83 (3.84/4.64)
Erlotinib	0.67	0.38	0.51 (2.79/5.49)	0.75 (6.89/9.19)	0.74	0.46	0.94 (5.15/5.50)
Flavopiridol	0.71	0.68	1.00 (3.09/3.09)		0.87	0.86	1.00 (3.09/3.09)
GW-2580	0.87	0.01	0.00 (0.00/255.80)		0.30	0.00	0.00 (0.00/256.20)
GW-786034	0.70	0.58	0.94 (5.14/5.49)		0.75	0.53	0.84 (4.60/5.45)
Gefitinib	0.65	0.29	0.37 (3.38/9.27)		0.55	0.11	0.00 (0.00/9.28)
Imatinib	0.63	0.21	0.38 (4.51/11.84)	0.25,0.50 (2.99,5.98/11.95)	0.63	0.22	0.55 (6.49/11.86)
JNJ-7706621	0.81	0.75	0.59 (1.17/2.00)		0.85	0.87	1.00 (2.00/2.00)
LY-333531	0.85	0.55	0.65 (4.57/7.03)		0.90	0.55	0.80 (5.61/7.04)
Lapatinib	1.00	0.99	0.86 (20.30/23.69)	0.00 (0.00/19.92)	0.71	0.07	0.00 (0.00/23.72)
MLN-518	0.87	0.24	0.16 (3.44/21.68)		0.75	0.28	0.20 (4.41/21.71)
MLN-8054	0.72	0.57	0.73 (4.99/6.84)		0.79	0.60	0.97 (6.64/6.85)
PI-103	0.75	0.11	0.00 (0.00/16.40)		0.93	0.30	0.30 (4.75/16.01)
PKC-412	0.49	0.40	0.00 (0.00/2.20)		0.81	0.71	0.53 (1.17/2.20)
PTK-787	0.95	0.22	0.22 (7.68/34.57)		1.00	0.99	0.58 (20.02/34.62)
Roscovitine	0.92	0.78	0.97 (4.66/4.81)	1.00 (2.81/2.81)	1.00	1.00	1.00 (4.82/4.82)
SB-202190	0.88	0.71	0.92 (3.90/4.24)		0.91	0.79	0.97 (4.08/4.21)
SB-203580	0.78	0.54	0.60 (2.24/3.71)	1.00 (5.43/5.43)	0.84	0.68	0.87 (3.23/3.69)
SB-431542	1.00	0.98	0.41 (20.30/49.19)		0.44	0.02	0.00 (0.00/49.27)
SU-14813	0.68	0.43	0.27 (0.83/3.08)		0.87	0.78	1.00 (3.09/3.09)
Sorafenib	0.82	0.62	0.87 (3.58/4.10)		0.63	0.48	0.89 (3.63/4.08)
Staurosporine	0.83	0.96	0.97 (1.11/1.14)		0.93	0.99	1.00 (1.15/1.15)
Sunitinib	0.70	0.48	0.21 (0.52/2.51)		0.87	0.83	1.00 (2.52/2.52)
VX-680	0.77	0.63	0.56 (1.64/2.95)		0.79	0.68	0.94 (2.77/2.95)
VX-745	0.87	0.47	0.48 (3.02/6.33)		0.49	0.19	0.36 (2.28/6.34)
ZD-6474	0.90	0.77	0.95 (4.30/4.52)		0.90	0.81	1.00 (4.53/4.53)
mean	0.80	0.55	0.59		0.76	0.54	0.66

For each of the 38 inhibitors in the affinity dataset of Karaman et al. [31], the prediction performance of ccorps, the Jackson et al. [9] method, and the sequence-based method is shown below. The performance of the Jackson et al. [9] method is shown alongside that of ccorps for the subset of inhibitors tested by both methods. Note that for imatinib, two 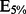 values are provided by Jackson et al. [9] because each value is derived by selecting a different reference structure. While the mean auc values and enrichment scores are close, the standard deviations of the *differences* between the corresponding columns (0.21, 0.33, and 0.36, respectively) highlight that the two methods have complementary strengths.

In order to make a direct comparison of the performance of ccorps to the work of Jackson et al. [9], another performance measure, the enrichment factor, was also computed per inhibitor tested. The enrichment factor of the top 5% most highly ranked true affinity predictions (for a given inhibitor) can be calculated as follows:
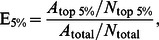
where 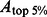 is the number of structures with known affinity for a given inhibitor (# *actives*) in the top 5% of most confident predictions ranked by 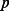-value as computed by ccorps, 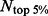 is the total number of structures in the top 5%, 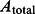 is the total number of active structures in the dataset and 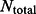 is total number of structures in the dataset. The enrichment factor at 5% (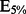) for each inhibitor is shown in Table 3 and where available, the corresponding 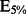 values from Jackson et al. [9] are listed alongside. It should be noted that the 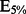 values are *not directly* comparable between ccorps and Jackson et al. [9] as listed in Table 3, due to the fact that the maximum possible enrichment (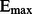) for a given inhibitor is dataset-dependent, and the dataset presented in this work is larger both in number of structures compared and the number of per-inhibitor affinity annotations. The ratio of 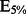 to 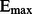 is a slightly better basis for comparison, since it normalizes for differences in 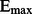.

As shown in Table 3, ccorps achieves high predictive performance across the 38 inhibitors tested. As quantified by roc auc 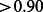, ccorps achieved perfect or near-perfect predictive ability for 8 of the 38 inhibitors: AMG-706, CHIR-265, CP-690550, CP-724714, Lapatinib, PTK-787, Roscovitine and SB-431542. Furthermore, ccorps is demonstrated to be very competitive with the method by Jackson et al. [9] as also shown in Table 3. In the case of Lapatinib, ccorps significantly improved on Jackson et al. [9]. Comparison of enrichment scores ignores another important difference: with our method no reference structure needs to be selected. As is clear from Jackson et al.'s result for imatinib, the 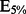 enrichment value can change by a factor of 2 depending on which reference structure is chosen.

Finally, in order to evaluate the contribution of the local structural features over sequence information alone, a “binding site sequence”-based approach was implemented (see *Text S3*) and used to predict inhibitor binding for the full 38 inhibitor dataset presented here. The prediction performance for the binding site sequence-based approach is shown in Table 3 in terms of roc and pr auc as well as enrichment score. The binding site sequence-based approach outperformed ccorps by a significant margin for several inhibitors: Staurosporine, Sunitinib, SU-14813, PKC-412, JNJ-7706621, VX-680. These 6 inhibitors on which ccorps significantly underperforms are 6 of the top 7 inhibitors in terms of number of kinases inhibited. That is, the aforementioned 6 inhibitors are relatively promiscuous and tend to interact with a large number of kinases across several kinase families. Furthermore these same 6 inhibitors also have the 6 highest hpc counts across the entire dataset. This result indicates that ccorps has difficulty predicting inhibitor binding for broad spectrum inhibitors and is discussed further in the following section. In the cases of JNJ-7706621 and VX-680, the performance difference is only significant in terms of the enrichment score. ccorps significantly outperforms the binding site sequence-based approach for several narrow spectrum inhibitors including, but not limited to: Lapatinib, CP-724714, CP-690550, SB-431542, VX-745. Overall, when ccorps performed better than the sequence-based approach the magnitude of the performance difference tended to be larger than when it performed worse. It should also be noted that the standard deviations of the *differences* between in 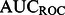, 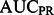, and enrichment score (0.21, 0.33, and 0.36, respectively) highlight that the two methods have complementary strengths. We repeated the cross-fold validation using 50% sequence identity (instead of 70%) to determine the folds. This makes the prediction problem harder for both ccorps and the sequence-based approach (see Table S2). The mean performance is slightly lower for both, but the standard deviations of the differences in performance remain the same, reinforcing the observation that ccorps and the sequence-based approach have complementary strengths.

## Discussion

Identifying structural features of the kinase binding site that directly or indirectly mediate the binding ability of inhibitors is a significant component in developing and optimizing kinase inhibitors. Given the increasingly large number of available kinase structures, kinome-wide comparative binding site analysis is now possible as has been demonstrated here. By combining available structure data with large-scale inhibitor affinity data, it becomes possible to automatically learn the features of the kinase binding site that predict the binding ability of a given inhibitor. This is useful for predicting whether kinases whose binding affinity is unknown will bind to a given drug, but, perhaps more importantly, knowing the structural basis for binding to a particular drug can be exploited in the design of analogs that bind more strongly and have fewer off-target interactions. This information could further improve well-established structure-based computer-aided drug design methods, where it is challenging to develop reliable models for the contributions of individual interactions or groups of interactions between inhibitor and protein to binding affinity.

ccorps has been demonstrated here to be capable of learning the features of the kinase binding site that are informative of inhibitor binding across a set of 38 inhibitors. Furthermore, the binding site features selected by ccorps as informative of inhibitor activity/inactivity have been shown to be interesting in and of themselves, for example, the existence of residue triad clusters that are unique in kinases capable of binding a given inhibitor but that exist within kinases from different major branches of the kinase family tree. The identification of such shared binding site features among sequence-diverse kinases is an important contribution for structure-based methods because of the relative difficulty of identifying small subsets of sequence non-contiguous but spatially compact positions that are correlated with a given indicator, such as inhibitor binding ability. The complete set of 41,964 true-majority HPCs that contain kinases from two or more of the kinome families as defined by Manning et al. [2] is provided as *Dataset S1* to facilitate further analysis of these phylogenetically diverse structural features that distinguish kinases binding each of the 38 inhibitors.

As was demonstrated here, ccorps is capable of incorporating all of the available protein kinase structure data, so as to operate at the kinome scale, and then using this data to construct highly accurate predictors of kinase affinity for a variety of different small molecule inhibitors. While ccorps relies upon the aggregation of structural similarity that coincides with affinity similarity to build predictors, the individual instances may be informative in and of themselves. Further analysis of the vast number of structurally similar features shared among phylogenetically distant kinases may provide additional insights into the structural mechanisms of inhibitor recognition occurring across the kinome.

The existence of affinity datasets containing structurally similar inhibitors, that differ by only one or a small number of chemical substitutions, provides the opportunity to associate specific structural features identified by ccorps with specific inhibitor pharmacophores. A recent approach by Milletti and Hermann [6] has been demonstrated to identify specific chemical transformations that can be associated with selectivity differences. In future work we will seek to further incorporate this cross-inhibitor level of analysis and broaden the scale of the structure dataset by further incorporating newly available kinase crystallographic structures.

Several potential optimizations of ccorps may increase its inhibitor binding prediction performance on broad spectrum inhibitors. For the 38 inhibitor dataset analyzed in this paper, the number of HPCs identified was well correlated with the number of kinases inhibited (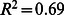). That is, ccorps tended to perform less well on inhibitors for which large numbers of HPCs were identified. Developing an approach to weighting and ranking the large number of HPCs generated by broad spectrum inhibitors may aid in increasing the predictive performance of ccorps for these inhibitors. For example, ranking HPCs by the mean within-cluster affinity (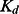) would more heavily weight structural features correlated with strong binders and decrease the impact of structural features only correlated weak binders. Such an approach would help to increase the signal-to-noise ratio of HPCs when the number of HPCs identified grows large. As our results showed, there are cases where ccorps significantly outperforms a sequence-based method, but there also cases where the reverse is true. While this paper focused on quantifying the extent at which structure alone can be used to predict binding affinity, for practical usage we envision that structure- and sequence-based methods are used in tandem.

A major advantage of the work presented is the generality of ccorps to detect structurally distinguishing features for a wide variety of applications beyond the kinase inhibitor affinity analysis presented here. No assumptions regarding the nature of the annotation labels nor of the alignment type are made at any point by ccorps. ccorps provides a general framework for automatically learning structural features that distinguish proteins having different annotation labels. This allows the incorporation of purely structure-based alignments, such as those available in databases like homstrad [33] or even local structure alignments such as those identified by motif/template search algorithms (e.g., soippa [34], and LabelHash, [35]). Other sources of annotation labels, including Gene Ontology (GO, [14]) terms, binding affinity for a given molecule and ligation state can be incorporated as-is with ccorps without modification to the method.

## Supporting Information

Figure S1
**Structure-based binding site alignment via MATT.** In order to identify a mapping between residues in the tk and non-tk Pfam alignments, matt was used to compute a structural alignment of the kinase domains of p38 structure pdb:3hec (white) and lck structure pdb:2pl0 (black), both with bound imatinib inhibitor (red). The 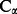 rmsd of the above binding site alignment region (27 residue positions) was 1.169 Å and the RMSD of the imatinib inhibitors is 1.736 Å; the imatinib molecule coordinates were ignored during computation of the alignment.(TIF)

Figure S2
**Substructure clustering for one 3-position subset of the** 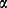 **-amylase binding site alignment.** In each scatter plot above, the dimensionality-reduced feature vectors computed by ccorps are shown. Each point shown is one feature vector and each feature vector represents one protein substructure. Tightly grouped points correspond to binding site substructures with high structural and chemical similarity. Plots A, B and C above all show the same clustering with different sets of annotation labels applied (labels are denoted by color): (A) cluster ID labeling; (B) 3-tier EC labeling; (C) 4-tier EC labeling. Solid ellipses indicated clusters identified automatically as HPCs. Dashed ellipses indicate subsets of non-hpc clusters that would have been considered HPCs if the clustering step had distinguished each as a separate cluster.(TIF)

Dataset S1 **The complete set of 41,964 true-majority HPCs that contain kinases from two or more of the kinome families.**
(CSV)

Text S1 **Details on the mapping and alignment of positions among structures within the kinase dataset.**
(PDF)

Text S2 **Using ccorps to predict the EC classification for an extensive dataset of 48 families constructed using the Pfam database: a large-scale validation experiment.**
(PDF)

Text S3 **Details of the ccorps method.**
(PDF)

Table S1 **Accuracy of predicted EC classifications for Pfam protein families in cross-fold validation.** Predictions are made at all 4 tiers of the EC hierarchy.
(PDF)

Table S2 **Affinity prediction performance of ccorps for the kinase inhibitors using a 50% sequence identity clusters for cross validation.** For each of the 38 inhibitors in the affinity dataset of Karaman et al., the prediction performance of ccorps and the sequence-based method is shown below. While the mean auc values and enrichment scores are close, the standard deviations of the *differences* between the corresponding columns (0.21, 0.31, and 0.40, respectively) highlight that the two methods have complementary strengths.
(PDF)
