# Supplementary figures and images for: Combinatorial Clustering of Residue Position Subsets Predicts Inhibitor Affinity across the Human Kinome
Source: PLoS Comput Biol. 2013 Jun 6;9(6):e1003087. doi: 10.1371/journal.pcbi.1003087 (PMC3675009; doi:10.1371/journal.pcbi.1003087)

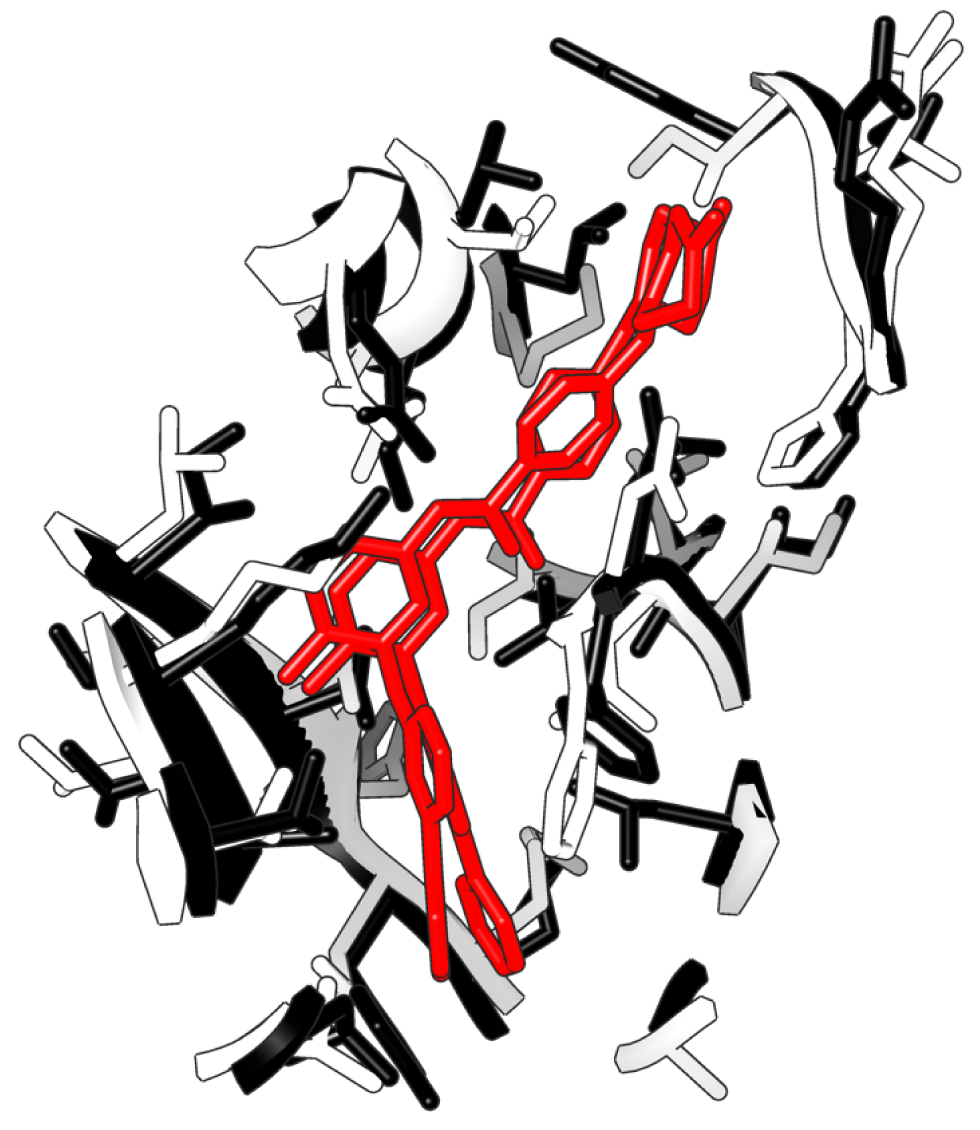

Supplement: Figure S1 — Structure-based binding site alignment via MATT. In order to identify a mapping between residues in the tk and non-tk Pfam alignments, matt was used to compute a structural alignment of the kinase domains of p38 structure pdb:3hec (white) and lck structure pdb:2pl0 (black), both with bound imatinib inhibitor (red). The rmsd of the above binding site alignment region (27 residue positions) was 1.169 Å and the RMSD of the imatinib inhibitors is 1.736 Å; the imatinib molecule coordinates were ignored during computation of the alignment. (TIF) [file pcbi.1003087.s001.tif]

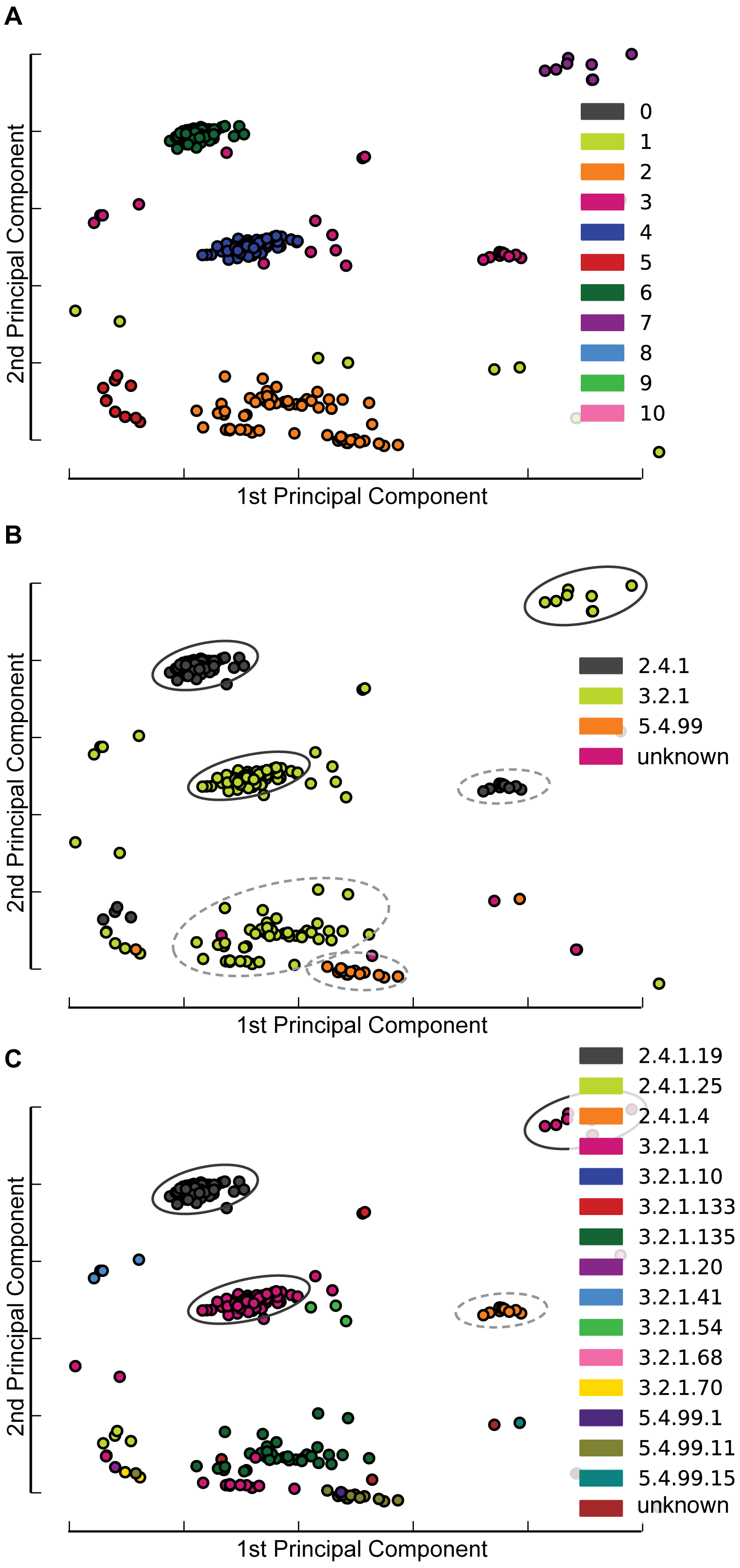

Supplement: Figure S2 — Substructure clustering for one 3-position subset of the -amylase binding site alignment. In each scatter plot above, the dimensionality-reduced feature vectors computed by ccorps are shown. Each point shown is one feature vector and each feature vector represents one protein substructure. Tightly grouped points correspond to binding site substructures with high structural and chemical similarity. Plots A, B and C above all show the same clustering with different sets of annotation labels applied (labels are denoted by color): (A) cluster ID labeling; (B) 3-tier EC labeling; (C) 4-tier EC labeling. Solid ellipses indicated clusters identified automatically as HPCs. Dashed ellipses indicate subsets of non-hpc clusters that would have been considered HPCs if the clustering step had distinguished each as a separate cluster. (TIF) [file pcbi.1003087.s002.tif]
