# Supplementary material for: Combinatorial Clustering of Residue Position Subsets Predicts Inhibitor Affinity across the Human Kinome
Source: PLoS Comput Biol. 2013 Jun 6;9(6):e1003087. doi: 10.1371/journal.pcbi.1003087 (PMC3675009; doi:10.1371/journal.pcbi.1003087)
Supplement: Text S1 — Details on the mapping and alignment of positions among structures within the kinase dataset. (PDF) [file pcbi.1003087.s004.pdf]

Text S1: Supporting Information

Combinatorial clustering of residue position subsets predicts inhibitor affinity across the human kinome

Drew H. Bryant, Mark Moll, Paul Finn, and Lydia E. Kavraki

Kinome dataset preparation: eukaryotic protein kinase alignment

In the case of the ePKs, both the non-TKs (PFAM:Pkinase) and TKs (PFAM:Pkinase_Tyr) were combined into a single, comprehensive structural dataset in order to provide structural coverage for the full kinase family tree. However, determining an appropriate residue position correspondence *between* the TK and non-TK family alignments requires an additional alignment step, in order to relate columns from the TK alignment to columns of the non-TK alignment. Several approaches for obtaining a consistent and high-quality alignment between the TKs and non-TKs were considered, such as profile-profile alignment and structure-based alignment.

To provide a structure-based solution to determining a high-quality residue position correspondence between the TKs and non-TKs, MATT [1] (version 1.00) was selected due to its ability to focus the alignment on regions of structural similarity (e.g., the ATP binding site) while disregarding regions with low structural similarity (e.g., C-terminal region). The kinase domain of non-TKs and TKs was then aligned using MATT by structural superposition of a pair of representative structures (PDB:3HEC and PDB:2PL0, respectively), that had both been co-crystallized with the same ATP binding site inhibitor (imatinib). The alignment RMSD of the common core region (220 residues) identified by MATT was 2.156 Å; the RMSD of the bound imatinib molecules was 1.736 Å. The MATT alignment is shown for the binding site residue positions analyzed here in Figure S1; the C_α RMSD of the 27 binding site residues shown is 1.169 Å. The aligned computed by MATT is shown below.

```
Core Residues: 220
Core RMSD: 2.156

3HEC:A   R--------------------PTFYRQELNKTIWEVPERYQNLSPVGSGAYG-----SVC    39  (A)
2PL0:A   -GSHMQTQKPQKPWWEDEWEVP--------------RETLKLVERLG-----AGQFGEVW   260  (B)

3HEC:A   AAFDTKTGLRVAVKKLSRPFQSII--HAKRTYRELRLLKHMKHENVIGLLDVFTPARSLE    97  (A)
2PL0:A   MGYYNG-HTKVAVKSLKQ--G---SMSPDAFLAEANLMKQLQHQRLVRLYAVVTQ-----   309  (B)

3HEC:A   EFNDVYLVTHLM-GADLNNIVKC---QKLTDDHVQFLIYQILRGLKYIHSADIIHRDLKP   153  (A)
2PL0:A   --EPIYIITEYMENGSLVDFLKTPSGIKLTINKLLDMAAQIAEGMAFIEERNYIHRDLRA   367  (B)

3HEC:A   SNLAVNEDCELKILDFGLARHTDDEMTGYVA-------------------TRWYRAPEIM   194  (A)
2PL0:A   ANILVSDTLSCKIADF---------------GLARLIEDNEYTAREGAKFPIKWTAPEAI   412  (B)

3HEC:A   LNWMHYNQTVDIWSVGCIMAELLTG-RTLF--PGTDHIDQLKLI--LRLVGTPGAELLKK   249  (A)
2PL0:A   NYGT-FTIKSDVWSFGILLTEIVTHGRIPYPGMTNP--EVIQNLERGYR-----------   458  (B)

3HEC:A   ISSESARNYIQSLTQMPKMNFA-NVFIGANPLAVDLLEKMLVLDSDKRITAAQALAHAYF   308  (A)
2PL0:A   ----------------------MVRPDNCPEELYQLMRLCWKERPEDRPTFDYLRSV-LE   495  (B)

3HEC:A   AQYHDPDDEPVADPYDQSFESRDLLIDEWKSLTYDEVISFVPPP-------------   352  (A)
2PL0:A   D-------------------------------------------FFTATEGQYQPQP   509  (B)
```

References

1. Menke M, Berger B, Cowen L (2008) Matt: local flexibility aids protein multiple structure alignment. PLoS Comput Biol 4: e10.
