# Supplementary material for: Combinatorial Clustering of Residue Position Subsets Predicts Inhibitor Affinity across the Human Kinome
Source: PLoS Comput Biol. 2013 Jun 6;9(6):e1003087. doi: 10.1371/journal.pcbi.1003087 (PMC3675009; doi:10.1371/journal.pcbi.1003087)
Supplement: Text S2 — Using ccorps to predict the EC classification for an extensive dataset of 48 families constructed using the Pfam database: a large-scale validation experiment. (PDF) [file pcbi.1003087.s005.pdf]

# Text S2: Supporting Information

**Combinatorial clustering of residue position subsets predicts inhibitor affinity across the human kinome**

Drew H. Bryant, Mark Moll, Paul Finn, and Lydia E. Kavraki

## 1 Pfam+EC benchmark experiments

After introducing the Pfam binding site dataset automatically constructed to benchmark CCORPS, we will introduce three distinct results of the method. First, we will discuss the accuracy of CCORPS in predicting EC classifications in a large-scale, cross-fold validation experiment. Second, we will demonstrate that HPCs are capable of distinguishing structures with differing EC classifications and that multiple HPCs can exist for a given EC class. We conclude this section with a discussion of the identification of specificity determining positions from HPCs.

The procedure implemented to automatically generate the Pfam binding site alignment dataset is outlined in Section 1.2. The two inputs to CCORPS are a structure alignment and a set of corresponding annotation labels (one label per structure). First, CCORPS computes structural clusterings combinatorially across all 3-position subsets of the binding site positions in order to search for structural features that distinguish binding sites with different annotation labels. For each specificity determining structural feature that is identified the positions responsible for the specificity are tallied, as described in the section *Selecting HPCs* in the main text. The alignment positions are finally ranked by their frequency of appearance in specificity-determining structural features.

### 1.1 EC Annotation labels

For each protein structure in a family, several different annotation labels are generated based upon the 4 tiers of EC classification. For example, a given structure with an EC classification of the form `A.B.C.D` can be labeled for each tier of the EC as `A.B.C.D`, `A.B.C.`$*$, `A.B.`$*.*$, or `A.`$*.*.*$. The 4-tiered label (`A.B.C.D`) provides a more precise functional label than the 1-tiered version (`A.`$*.*.*$). The objective is to predict all 4 EC labels for structures with unknown EC classification.

### 1.2 Automated binding site definition

All alignments used in this work were derived from Pfam MSA s [1]. A Pfam MSA provides an alignment of homologous protein domains. For each aligned domain in an MSA, the UniProt [2] ID is retrieved from the Pfam alignment and all PDB [3] structures corresponding to the given UniProt ID are mapped to the domain sequence. The Pfam MSA alignment column positions define the mapping of residue positions across all structures for a protein family.

#### 1.2.1 Selecting binding site positions

For each aligned structure that contains one or more non-protein molecules (distinguished by HETATM records) with $\geq 30$ atoms, the largest available molecule was identified and assumed to be a ligand. For each ligated structure, all residues having at least one atom within 5Å of one or more ligand atoms were selected as potential binding site residues. These binding site residues were then mapped to columns within the Pfam MSA. A count is kept for the number of times each MSA column was mapped to a residue in a ligated structure. After tabulating MSA column mapping counts across all ligated structures, only MSA columns that were mapped to binding site residues in $\geq 5$ instances were retained.

#### 1.2.2 Identifying a dense sub-alignment

Next, it is necessary to remove gaps from the input alignment so that all pairwise comparisons of binding site positions are consistent, as outlined in the section *Calculating feature vectors* in the main text. When gaps appear in the aligned binding site column positions, either the entire column position must be eliminated from further analysis or all protein structures having a gap at the alignment position must be eliminated. This "densification" procedure of removing either a gapped row (protein structure) or gapped column (alignment site position) is repeated until only a fully "dense" (non-gapped) sub-matrix remains. In the resulting dense sub-matrix, all remaining protein structures have a residue at all remaining alignment positions.

Finding the largest dense sub-matrix in the alignment as outlined above is equivalent to finding a maximal edge biclique in a bipartite graph. Given a graph $G = (V_1 + V_2, E)$, alignment positions are vertices in $V_1$ and protein structures are vertices in $V_2$. Each non-gapped position for a protein $v_i \in V_1$ at a position $v_j \in V_2$ in the alignment is the edge $E = v_1 v_2$. Identifying the maximal biclique in such a bipartite graph has been shown to be NP-complete [4].

The heuristic densification approach implemented to identify dense sub-matrices of alignments is as follows. (1) Given $n$ structures aligned (with gaps) at $m$ positions, convert the alignment to an $n \times m$ binary matrix $M$ such that $M[i][j] = 0$ if structure $i$ was gapped in the alignment at position $j$ and $M[i][j] = 1$ otherwise. (2) Consider each row $M[i]$ to be a binary vector representing structure $i$. (3) Compute the complete-linkage hierarchical clustering [5] of the binary vectors using the Hamming distance metric [6]. (4) Each node of the resulting hierarchical clustering represents one potential sub-matrix. Calculate the dense size of the sub-matrix by removing all rows or columns containing one or more zeros from the sub-matrix and taking the sum of the remaining values. (5) Select the sub-matrix with maximal dense size. Note that the maximal dense sub-matrix selected in step (5) is not guaranteed to be the optimal sub-matrix because every possible sub-matrix of the original matrix does not exist as a node in the hierarchical clustering. The rows (protein structures) and columns (alignment positions) for the selected dense sub-matrix are used to prune the raw alignment positions and structures in order to provide a fully dense "sub-alignment" as input to CCORPS.

Techniques used for finding dense sub-matrices within real-valued gene expression data such as "biclustering" are potential alternatives to the heuristic approach used here (see [7] for a review of biclustering approaches). However, the binding site position subset of an alignment is often quite dense, making the sparseness assumptions of gene expression biclustering methods unessential for the current Pfam alignment dataset.

### 1.3 Dataset

#### 1.3.1 Selection of families from PFAM

All 12,273 protein families from the Pfam 25.0 release (April 2011) were considered for inclusion in the dataset. Only protein families that met the following criteria were selected for inclusion: $\geq 200$ domain structures; $\geq 10$ unique sequences, $\geq 2$ distinct EC classes; for the subset of sequences with known EC class, $\geq 2$ sequences from each of $\geq 2$ EC classes, all having $\leq 50\%$ sequence identity. Our dataset consists of the 48 protein families that meet or exceed these criteria. The criteria were chosen so that there would be enough structural and sequence diversity to make the prediction of EC classifications sufficiently challenging. With these criteria, the whole prediction process can (and has been) completely automated. No manual tuning was done to account for binding site size, EC class size, number of structures, etc.

#### 1.3.2 Dataset statistics

The protein family dataset that we have constructed in the manner described above covers a wide range of families with very different levels of functional diversification and binding site sizes as shown in Table S1.

The mean number of unique EC classes across families in the dataset was 8.3, with some families having as few as 3 different EC classes and as many as 21. An even wider variance is seen for the number of structures available per family, ranging from as few as 11 to as many as 548 with a mean of 108 for the dataset. Finally, the number of binding site positions examined ranged from the minimum of 3 to as many as 81 (mean of 33), covering a large range in binding site sizes.

## 1.4 Prediction performance

The performance of CCORPS was evaluated by applying the cross-validation procedure outlined in the main text to each of the protein families in the Pfam alignment dataset. For each protein family, the ability of CCORPS to predict enzymatic function annotation labels in the form of EC class numbers was quantified. The prediction accuracy of CCORPS for predicting EC classification at each of the 4 tiers of EC specificity is show in Table S1.

Due to the hierarchical nature of the EC classification system, the number of unique 4-tier EC classes (most specific annotation labels) for a family is necessarily greater than or equal to the number of unique 1-tier EC classes (least specific annotation labels). As can be noted by examining the dataset mean prediction accuracy from 1-tier to 4-tier, accuracy decreases with increasing EC classification annotation label specificity, as should be expected. The prediction accuracy at the least specific 1-tier EC classification was $92 \pm 18\%$, while the accuracy dropped to $53 \pm 30\%$ for the most specific 4-tier. With these numbers one needs to consider the very general automated procedure used to specify the input (e.g., the way binding site residues were chosen).

A major challenge when attempting to predict the 4-tier (most specific) EC annotation labels derives from the non-uniformity in structure coverage across the EC labels within a protein family. What could be considered "outlier" EC classes with only a single corresponding protein sequence within a protein family, were common throughout the dataset. Given the stringent cross validation procedure used in this work, it is actually impossible to correctly predict the annotation label for single-protein EC classes. This is due to the fact that when the structures for the single-protein EC class fall within the test set during one fold of the cross validation, no structures will exist in the training set that share the same EC class label by definition of the NR-clusters We chose to be conservative and not correct for this self-penalizing aspect of our performance benchmarking. The reason for this is that it reflects the realistic case of predicting enzymatic function for homologous proteins with unknown function that may be novel relative to the training dataset.

## 1.5 Highly predictive clusterings

The basis for the predictive ability of CCORPS is the detection of HPCs as outlined in the section *Selecting HPCs* in the main text. The set of clusters identified by CCORPS for one of the 2600 3-position subsets for the $\alpha$-amylase family is shown in Figure S2. Note that $\binom{26}{3} = 2600$, where 26 is the number of binding site positions available for the $\alpha$-amylase protein family as listed in Table S1.

In the subplots of Figure S2, the points shown each represent a feature vector (as described in the section *Calculating feature vectors* in the main text), where each feature vector corresponds to a single protein substructure. A tightly grouped cluster of feature vectors in the subplots of Figure S2 reflects a set of substructures sharing a high degree of structural and chemical similarity. Figure S2A shows the cluster membership automatically identified by CCORPS. Figure S2B and C show the EC annotation labels that map to each feature vector at the 3-tier and 4-tier levels, respectively. In the last two plots the points are colored by EC tier level rather than by cluster.

As can be seen in Figure S2B and C, several HPCs for different labels can exist simultaneously in a single clustering. Also, as shown in Figure S2B for the 3-tier EC label `3.2.1`, multiple distinct HPCs for a single label can be identified. In other words, within EC label `3.2.1` several structural sub-groups can be detected. The existence of distinct HPCs for a single label indicates that multiple structurally

and chemically distinct sub-groups can exist within a common annotation label. It is possible to identify such instances because CCORPS makes no assumptions about the structural homogeneity of sub-families having the same enzymatic function.

# References


1. Finn RD, Tate J, Mistry J, Coggill PC, Sammut SJ, et al. (2008) The Pfam protein families database. Nucleic Acids Res 36: D281-8.

2. Magrane M, Uniprot Consortium (2011) Uniprot knowledgebase: a hub of integrated protein data. Database (Oxford) 2011: bar009.

3. Berman H, Westbrook J, Feng Z, Gilliland G, Bhat T, et al. (2000) The Protein Data Bank. Nucleic Acids Research 28: 235–242.

4. Peeters R (2003) The maximum edge biclique problem is np-complete. Discrete Applied Mathematics 131: 651–654.

5. Johnson S (1967) Hierarchical clustering schemes. Psychometrika 32: 241–254.

6. Hamming R (1980). Coding and information theory.

7. Madeira S, Oliveira A (2004) Biclustering algorithms for biological data analysis: a survey. Computational Biology and Bioinformatics, IEEE/ACM Transactions on 1: 24–45.
