# Supplementary material for: Combinatorial Clustering of Residue Position Subsets Predicts Inhibitor Affinity across the Human Kinome
Source: PLoS Comput Biol. 2013 Jun 6;9(6):e1003087. doi: 10.1371/journal.pcbi.1003087 (PMC3675009; doi:10.1371/journal.pcbi.1003087)
Supplement: Text S3 — Details of the ccorps method. (PDF) [file pcbi.1003087.s006.pdf]

Text S3: Supporting Information

Combinatorial clustering of residue position subsets predicts inhibitor affinity across the human kinome

Drew H. Bryant, Mark Moll, Paul Finn, and Lydia E. Kavraki

Method Details

Within CCORPS care needs to be taken of overrepresentation of protein sequences in our input data. This needs to be done at two different steps of the method: the dimensionality reduction step for computing a low-dimensional embedding of each protein substructure and the step of selecting HPCs. The specific corrections made in these two steps are described in detail below. This section concludes with a detailed description of the binding site sequence-based affinity prediction method and cross-validation experiment.

Correcting for overrepresentation bias in dimensionality reduction

We use PCA to obtain a low-dimensional embedding of the *feature vectors*, i.e., rows in the matrix of pairwise distances between protein substructures. By default, PCA weights the importance of all feature vectors equally when computing the low-dimensional embedding that optimally preserves the variance of the original data. However, overrepresented protein sequences cause PCA to place unequal emphasis on preserving the variance among structures for the overrepresented sequence rather than equitably across all of the sequences in the dataset.

To remove overrepresentation bias, PCA is first computed with a non-redundant subset of the rows of the dissimilarity matrix; that is, one feature vector is computed for each 50% sequence identity cluster via selection of a representative structure for each sequence cluster (highest resolution structure is selected as representative). Then PCA is computed for this sequence non-redundant set of feature vectors alone, thereby avoiding the bias induced by structurally overrepresented sequences. Next, the feature vectors are computed for all structures in the dataset and then transformed to a lower dimensional embedding using the PCA transformation matrix computed from the non-redundant subset of the dataset. This approach amounts to computing a binary weighted PCA where all feature vectors have a weight of 0 with the exception of the non-redundant subset of structures that have a weight of 1.

Because of the large degree of structural overrepresentation for many proteins, it was possible to randomly sample a subset of the dissimilarity matrix columns and then apply the same dimensionality reduction and clustering steps while preserving the same cluster membership. This is in fact a common technique in dimensionality reduction [1]: to "localize" a protein it is sufficient to know the distance to small number of "landmark" proteins. The dissimilarity matrix column sampling procedure implemented partitions a protein family into sequence non-redundant groups at the 50% sequence identity level. Then, the highest resolution structure from l randomly selected non-redundant groups is selected. In this paper, $l = 20$ was used in all cases. The value of $l = 20$ was selected by parameter sweeping from $l = 1$ to $l = 50$ representatives using benchmarking experiments from our previous FASST dataset [2]; $l = 10$ was found to sufficiently reproduce the same structural clustering and $l = 20$ was chosen to be conservative. By randomly sampling columns from the dissimilarity matrix, the computation is reduced from $O(n^2)$ to $O(ln)$ where $l \ll n$ typically, allowing CCORPS to scale easily as additional structures become available.

Correcting for overrepresentation bias in HPC selection

In order to eliminate label bias in clusters due to overrepresentation, a sequence non-redundant version of cluster purity was implemented. Protein sequences were clustered for all structures at the 100% sequence identity level for determining non-redundant groups in this case. To adjust purity for structural

overrepresentation, NR-purity is calculated as NR-purity= $I_{L_{nr}}(mode(L_{nr}))$ where L_{nr} is one label from each NR $_{100}$ sequence identity cluster that exists within L.

Additionally, the purity of a set of labels must address the presence of proteins with unknown label in a cluster, which will occur because of the semi-supervised nature of CCORPS. In this work, proteins with unknown label have no effect on the purity calculated for a set of labels.

Binding site sequence-based benchmark comparison

In order to benchmark the predictive ability of CCORPS across the full set of 38 inhibitors available in the affinity dataset, a sequence-based method was implemented and applied to the same kinase dataset analyzed by CCORPS. The dataset used in this paper for the CCORPS experiments was also used without modification for the binding site sequence-based approach. In particular, the sequences being analyzed are already pre-aligned by the curated PFAM:Pkinase and PFAM:Pkinase_Tyr family alignments and the correspondence between the Pfam families determined by structural alignment as described in *Text S1*. The sequence identity of a pair of kinases is then the fraction of binding site positions with equivalent residues using the alignment described in *Text S1*.

Given the binding site sequence identity approach outlined above, the following procedure was performed for each of the 38 inhibitors within the dataset using the same cross validation procedure as CCORPS:

1. Compute one cross validation fold per sequence cluster as defined by CCORPS cross validation procedure. For each kinase sequence s_i in current cluster/fold C compute a binding confidence score as follows:

   Select the nearest neighbor sequence s'. The nearest neighbor s' for kinase s_i is the kinase sequence with the highest binding site sequence identity to s_i that is in the labeled training set (i.e., outside of the current fold):

   $$s' = \underset{s_j \in C}{\text{argmax}}\ \textsc{BindingSiteSequenceIdentity}(s_i, s_j) \text{ such that } s_j \notin C$$

   Predict label of s_i to be that of s'.

   Assign binding confidence score to prediction. The *binding confidence* score is computed as follows. If s' binds the inhibitor, then the score is set to the binding site sequence identity between s' and s_j, otherwise it is set to the negated binding site sequence identity.

2. Sort predictions by descending binding confidence score
3. Compute ROC and PR curves based on ranked predictions

References

1. Platt J (2005) Fastmap, MetricMap, and Landmark MDS are all Nyström algorithms. In: Proceedings of the 10th International Workshop on Artificial Intelligence and Statistics. Omni Press Madison, WI, pp. 261–268.

2. Bryant DH, Moll M, Chen BY, Fofanov VY, Kavraki LE (2010) Analysis of substructural variation in families of enzymatic proteins with applications to protein function prediction. BMC Bioinformatics 11: 242.
