# Supplementary material for: Combinatorial Clustering of Residue Position Subsets Predicts Inhibitor Affinity across the Human Kinome
Source: PLoS Comput Biol. 2013 Jun 6;9(6):e1003087. doi: 10.1371/journal.pcbi.1003087 (PMC3675009; doi:10.1371/journal.pcbi.1003087)
Supplement: Table S1 — Accuracy of predicted EC classifications for Pfam protein families in cross-fold validation. Predictions are made at all 4 tiers of the EC hierarchy. (PDF) [file pcbi.1003087.s007.pdf]

Table S1. Accuracy of predicted EC classifications for Pfam protein families in cross-fold validation. Predictions are made at all 4 tiers of the EC hierarchy.

	Family statistics			EC prediction accuracy (%)			
Pfam ID	#EC	#Struct.	Size[1]	1-tier	2-tier	3-tier	4-tier
4HBT	5	37	28	100	95	95	10
AAA	4	57	14	30	26	26	26
ADH_N	10	62	9	100	99	99	68
Aldedh	15	66	58	100	90	90	71
Alpha-amylase	16	220	26	89	89	89	71
Amino_oxidase	7	51	33	100	12	12	6
Aminotran_1_2	15	148	32	96	95	95	39
Asp	11	54	75	100	100	100	14
COesterase	6	152	62	100	100	100	78
Cu-oxidase	7	95	11	100	88	49	49
DHFR_1	5	155	81	94	94	94	92
ECH	10	38	43	81	79	79	36
Epimerase	9	62	71	95	95	95	88
Ferritin	4	79	15	90	90	2	2
GST_C	5	209	5	91	91	91	91
GST_N	4	134	18	98	98	98	98
Glyco_hydro_18	3	112	16	100	100	98	98
Gp_dh_C	4	47	11	100	100	100	88
Hexapep	13	76	13	90	75	75	48
Lactamase_B	9	119	4	98	44	40	40
Ldh_1_C	4	112	3	100	100	100	58
Ldh_1_N	4	64	43	100	100	100	86
Lys	8	548	22	100	100	100	96
NUDIX	9	33	41	90	88	85	24
PALP	18	113	9	72	52	52	44
PDEase_I	5	98	28	100	100	100	82
Peptidase_C1	11	100	54	99	99	99	16
Peptidase_C14	6	19	44	100	100	100	45
Peptidase_M10	12	131	64	100	100	99	30
Peptidase_S9	7	100	12	100	99	97	94
Pkinase	11	145	69	100	100	57	52
Pkinase_Tyr	5	142	66	100	100	66	52
Proteasome	3	20	64	100	100	46	46
Proteasome_A_N	3	21	7	100	100	47	47
Pyr_redox	21	145	14	100	65	65	57
Pyr_redox_2	11	49	76	100	59	59	10
Pyr_redox_dim	12	94	7	100	56	56	38
RVP	9	418	35	99	99	97	93
Ribonuclease	6	158	14	100	100	91	52
Rieske	5	63	33	100	96	96	74
TPP_enzyme_C	11	59	21	83	83	56	48
TPP_enzyme_N	14	78	17	76	81	52	42
Thioredoxin	12	171	3	44	0	0	0
Thymidylat_synt	5	106	45	92	92	92	86

Table S1. Accuracy of predicted EC classifications for Pfam protein families in cross-fold validation. Predictions are made at all 4 tiers of the EC hierarchy.

	Family statistics			EC prediction accuracy (%)			
Pfam ID	#EC	#Struct.	Size[1]	1-tier	2-tier	3-tier	4-tier
adh_short	21	137	65	100	72	72	35
efhand	14	308	17	15	15	1	0
p450	7	67	43	100	100	18	16
peroxidase	3	45	63	100	100	100	100
**Mean**				92	84	74	53
**Standard Deviation**				18	25	30	30
