# Supplementary material for: Combinatorial Clustering of Residue Position Subsets Predicts Inhibitor Affinity across the Human Kinome
Source: PLoS Comput Biol. 2013 Jun 6;9(6):e1003087. doi: 10.1371/journal.pcbi.1003087 (PMC3675009; doi:10.1371/journal.pcbi.1003087)
Supplement: Table S2 — Affinity prediction performance of ccorps for the kinase inhibitors using a 50% sequence identity clusters for cross validation. For each of the 38 inhibitors in the affinity dataset of Karaman et al., the prediction performance of ccorps and the sequence-based method is shown below. While the mean auc values and enrichment scores are close, the standard deviations of the differences between the corresponding columns (0.21, 0.31, and 0.40, respectively) highlight that the two methods have complementary strengths. (PDF) [file pcbi.1003087.s008.pdf]

Table S2. Affinity prediction performance of ccorps for the kinase inhibitors using a 50% sequence identity clusters for cross validation. For each of the 38 inhibitors in the affinity dataset of Karaman et al., the prediction performance of CCORPS and the sequence-based method is shown below. While the mean AUC values and enrichment scores are close, the standard deviations of the *differences* between the corresponding columns (0.21, 0.31, and 0.40, respectively) highlight that the two methods have complementary strengths.

	CCORPS			Sequence-based		
**Inhibitor**	AUC_{ROC}	AUC_{PR}	$E_{5\%}/E_{max}$	AUC_{ROC}	AUC_{PR}	$E_{5\%}/E_{max}$
ABT-869	0.52	0.14	0.19 (1.60/8.41)	0.50	0.27	0.44 (3.69/8.43)
AMG-706	0.87	0.66	0.83 (5.59/6.77)	0.67	0.48	0.81 (5.45/6.71)
AST-487	0.68	0.67	0.44 (0.76/1.71)	0.72	0.75	0.77 (1.31/1.71)
AZD-1152HQPA	0.52	0.21	0.44 (3.01/6.77)	0.58	0.29	0.42 (2.86/6.78)
BIRB-796	0.83	0.71	0.98 (3.23/3.28)	0.72	0.63	0.78 (2.55/3.27)
BMS-387032	0.87	0.74	0.90 (3.33/3.69)	0.92	0.87	1.00 (3.69/3.69)
CHIR-258	0.82	0.69	0.95 (3.85/4.05)	0.89	0.82	1.00 (4.05/4.05)
CHIR-265	0.93	0.68	0.67 (4.87/7.31)	0.85	0.78	1.00 (7.24/7.24)
CI-1033	0.60	0.42	0.63 (2.84/4.47)	0.71	0.56	0.91 (4.06/4.48)
CP-690550	0.95	0.55	0.29 (9.37/32.79)	0.55	0.46	0.28 (9.24/32.85)
CP-724714	1.00	0.99	0.86 (20.30/23.69)	0.63	0.05	0.00 (0.00/23.72)
Dasatinib	0.57	0.39	0.17 (0.51/2.90)	0.77	0.65	0.83 (2.39/2.89)
EKB-569	0.73	0.51	0.70 (3.24/4.63)	0.79	0.62	0.91 (4.21/4.64)
Erlotinib	0.55	0.28	0.46 (2.53/5.49)	0.75	0.53	0.87 (4.81/5.50)
Flavopiridol	0.67	0.47	0.70 (2.16/3.09)	0.79	0.63	0.47 (1.45/3.09)
GW-2580	0.44	0.00	0.00 (0.00/255.80)	0.20	0.00	0.00 (0.00/256.20)
GW-786034	0.78	0.53	0.75 (4.10/5.49)	0.57	0.40	0.66 (3.58/5.45)
Gefitinib	0.43	0.10	0.06 (0.59/9.27)	0.26	0.11	0.09 (0.87/9.28)
Imatinib	0.70	0.22	0.30 (3.57/11.84)	0.28	0.13	0.34 (4.08/11.86)
JNJ-7706621	0.66	0.60	0.37 (0.73/2.00)	0.75	0.76	0.88 (1.75/2.00)
LY-333531	0.85	0.45	0.62 (4.35/7.03)	0.77	0.36	0.30 (2.09/7.04)
Lapatinib	1.00	0.99	0.86 (20.30/23.69)	0.63	0.05	0.00 (0.00/23.72)
MLN-518	0.88	0.19	0.21 (4.47/21.68)	0.54	0.05	0.00 (0.00/21.71)
MLN-8054	0.74	0.51	0.65 (4.45/6.84)	0.82	0.68	1.00 (6.85/6.85)
PI-103	0.97	0.86	0.94 (15.36/16.40)	0.99	0.90	1.00 (16.01/16.01)
PKC-412	0.29	0.34	0.05 (0.10/2.20)	0.67	0.68	1.00 (2.20/2.20)
PTK-787	1.00	0.88	0.59 (20.30/34.57)	0.89	0.86	0.50 (17.31/34.62)
Roscovitine	0.84	0.41	0.06 (0.31/4.81)	0.38	0.18	0.28 (1.35/4.82)
SB-202190	0.84	0.74	0.94 (3.97/4.24)	0.88	0.78	0.98 (4.15/4.21)
SB-203580	0.69	0.51	0.60 (2.24/3.71)	0.81	0.69	0.75 (2.77/3.69)
SB-431542	1.00	0.87	0.41 (20.30/49.19)	0.91	0.10	0.00 (0.00/49.27)
SU-14813	0.54	0.32	0.06 (0.20/3.08)	0.83	0.79	1.00 (3.09/3.09)
Sorafenib	0.50	0.49	0.90 (3.71/4.10)	0.63	0.45	0.81 (3.31/4.08)
Staurosporine	0.90	0.98	1.00 (1.14/1.14)	0.80	0.97	1.00 (1.15/1.15)
Sunitinib	0.47	0.35	0.05 (0.12/2.51)	0.86	0.84	1.00 (2.52/2.52)
VX-680	0.76	0.76	0.95 (2.81/2.95)	0.70	0.64	0.72 (2.12/2.95)
VX-745	0.40	0.27	0.59 (3.72/6.33)	0.50	0.18	0.34 (2.18/6.34)
ZD-6474	0.78	0.64	0.83 (3.73/4.52)	0.86	0.77	1.00 (4.53/4.53)
mean	0.73	0.53	0.55	0.69	0.52	0.64
